# Supplementary material for: Phylogenetic conservatism drives nutrient dynamics of coral reef fishes
Source: Nat Commun. 2021 Sep 14;12:5432. doi: 10.1038/s41467-021-25528-0 (PMC8440548; doi:10.1038/s41467-021-25528-0)

Supplementary Information

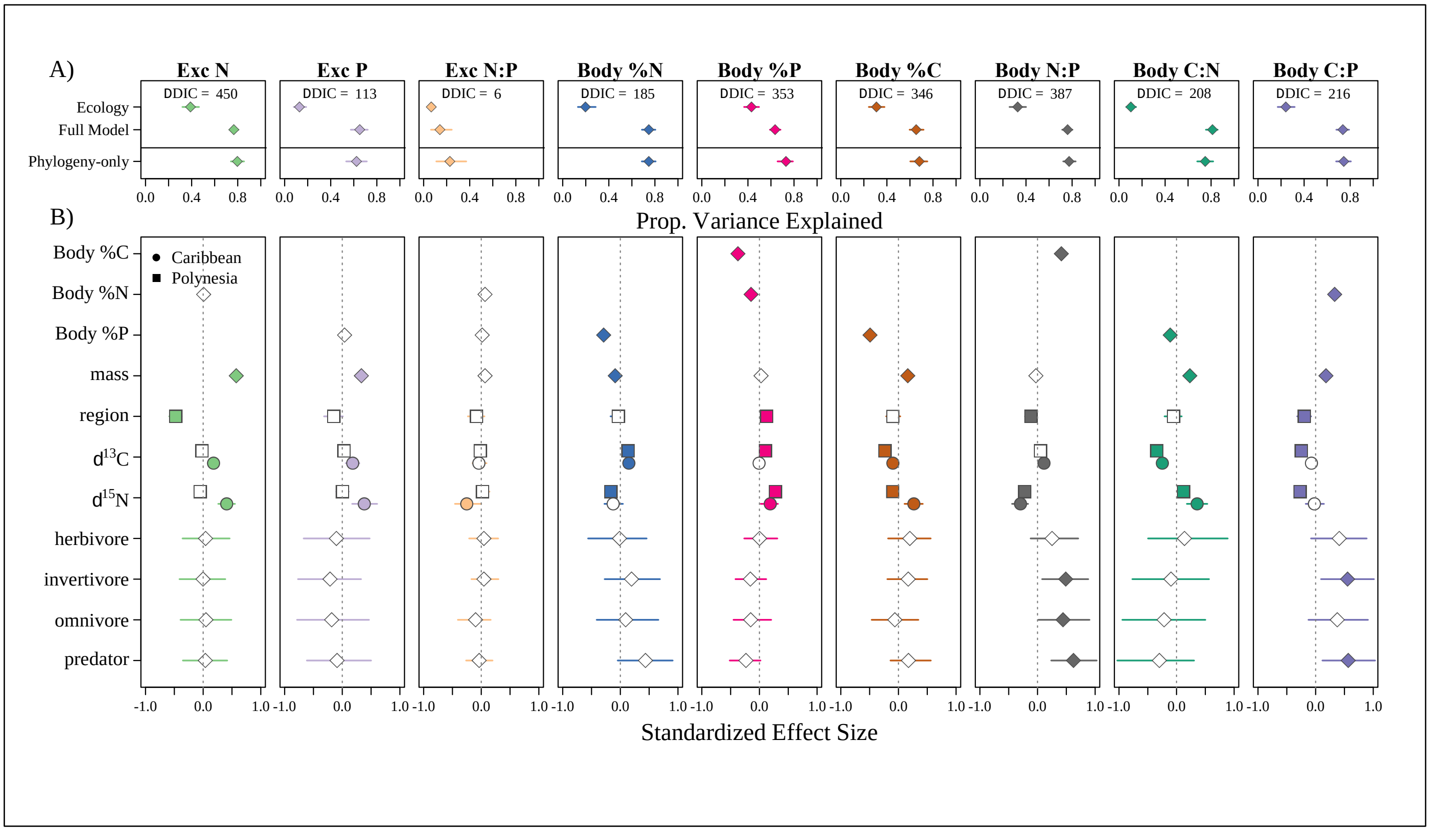

Supplementary Figure 1. Phylogenetic conservatism is the best predictor of chemical trait variation in coral reef fishes. This figure is the same as Figure 1 from the main text with the exception that Exc N and P are not mass corrected in the analyses used here. A) The variance explained by the ecological terms in the model (fixed effects), the full model (including fixed plus random effects), and the phylogeny-only models. Error bars indicate 95% Credible Intervals (CIs) associated with model error. ΔDIC indicates the best model with a value > 2 showing favor for the full model. B) Standardized effect sizes and CIs (diamonds) of all ecology variables in the model (all fixed effects). Error bars indicate 95% Credible Intervals (CIs) associated with model error. Body nutrient predictors (e.g., Body %P) are not in all models due to strong covariance among the variables, and were chosen as such to test specific hypotheses. Circles indicate estimates for the Caribbean fishes. Squares indicate estimates for Polynesian fishes and in the case of ‘region’ indicate if the chemical trait significantly differs from the Caribbean (the model intercept). Filled points indicate the CIs do not overlap with zero.

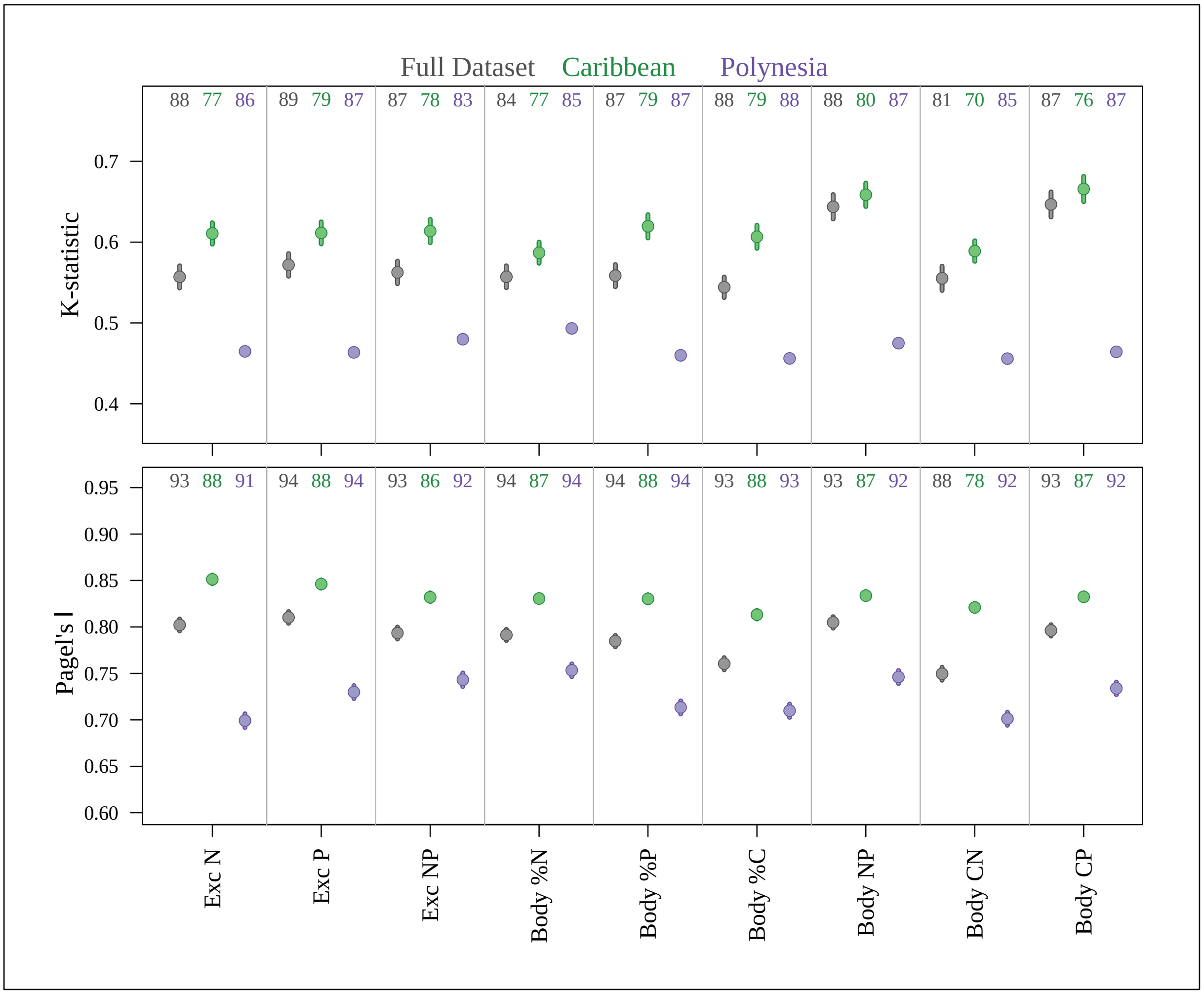

Supplementary Figure 2. Phylogenetic signal for each region (by color) and each trait (x-axis) as quantified by Bloomberg's K, and Pagel's Lambda. Values in the header of each plot indicate the percentage of bootstrap iterations that significantly differed from a randomly simulated community (see Main Text Methods).

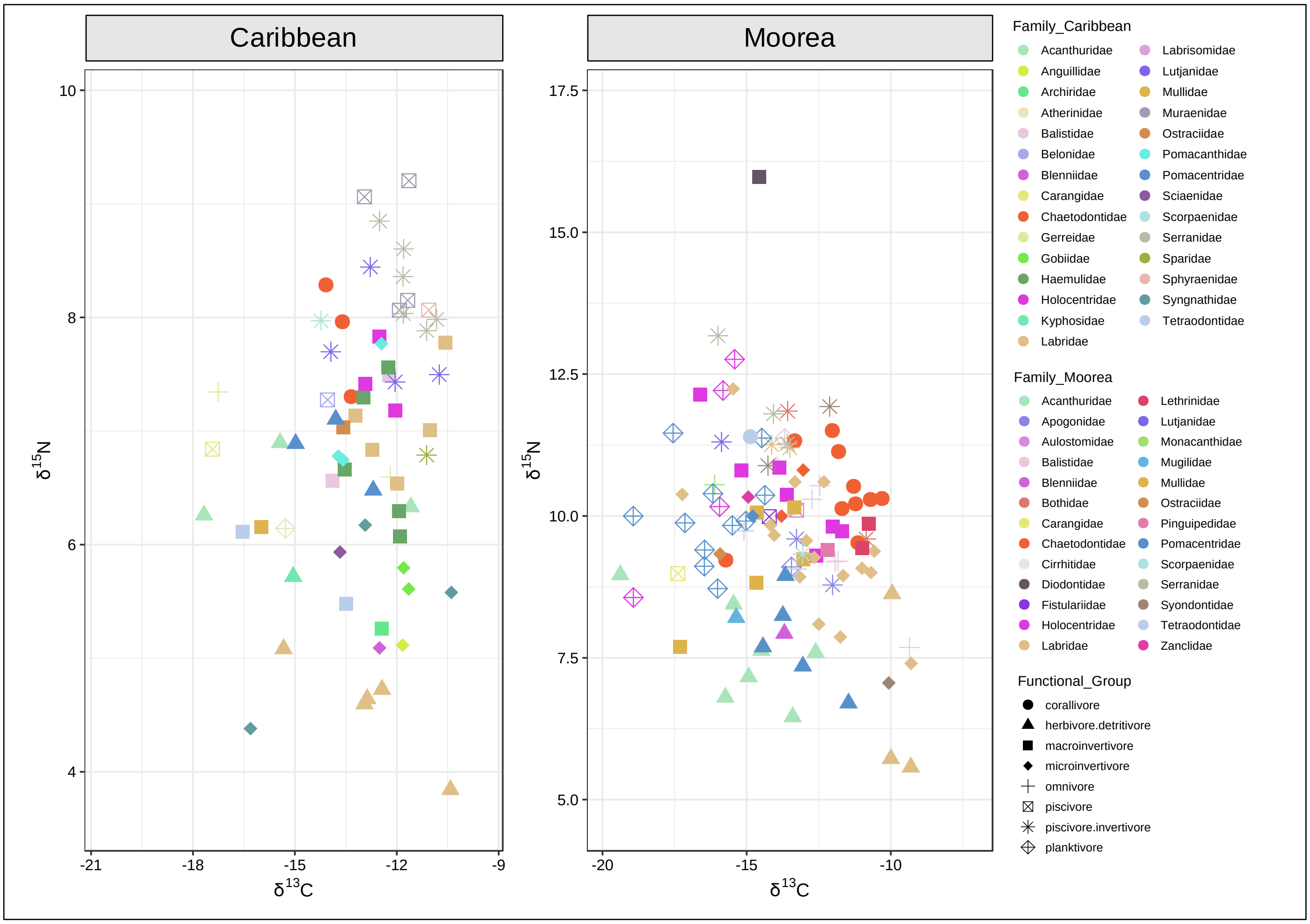

Supplementary Figure 3 – Isotopic Biplots for family-level means in each region (colored circles). Error bars indicate SD. Colors that are the same in both regions represent families that are shared across regions. Symbols indicate the Functional Group (or trophic groupings) used in our study.

Supplementary Figure 4 – Phylogenetic trees for all chemical traits with overlay of color scheme for values for The Bahamas

Supplementary Figure 5 – Phylogenetic trees for all chemical traits with overlay of color scheme for values for Mo'orea

Supplementary Table 1. All species and the number of individuals per species ('Count') used in our analysis. Included are the region, family and trophic grouping for each species.

Region	Family	Genus Species	count	FG
Caribbean	Pomacentridae	Abudefduf_saxatilis	9	herbivore.detritivore
Caribbean	Acanthuridae	Acanthurus_bahianus	2	herbivore.detritivore
Caribbean	Acanthuridae	Acanthurus_chirurgus	8	herbivore.detritivore

Caribbean	Acanthuridae	Acanthurus_coeruleus	7	herbivore.detritivore
Caribbean	Anguillidae	Anguilla_rostrata	2	microinvertivore
Caribbean	Atherinidae	Atherinomorus_stipes	7	planktivore
Caribbean	Balistidae	Balistes_capriscus	5	macroinvertivore
Caribbean	Balistidae	Balistes_vetula	2	macroinvertivore
Caribbean	Gobiidae	Bathygobius_soporator	18	microinvertivore
Caribbean	Sparidae	Calamus_nodosus	2	piscivore.invertivore
Caribbean	Tetraodontidae	Canthigaster_rostrata	8	macroinvertivore
Caribbean	Carangidae	Caranx_latus	1	piscivore
Caribbean	Chaetodontidae	Chaetodon_capistratus	21	corallivore
Caribbean	Chaetodontidae	Chaetodon_ocellatus	2	corallivore
Caribbean	Chaetodontidae	Chaetodon_striatus	6	corallivore
Caribbean	Gobiidae	Coryphopterus_dicrus	1	microinvertivore
Caribbean	Syngnathidae	Cosmocampus_albirostris	4	microinvertivore
Caribbean	Serranidae	Epinephelus_adscensionis	1	piscivore.invertivore
Caribbean	Serranidae	Epinephelus_guttatus	5	piscivore.invertivore
Caribbean	Serranidae	Epinephelus_morio	1	piscivore.invertivore
Caribbean	Serranidae	Epinephelus_striatus	21	piscivore.invertivore
Caribbean	Gerreidae	Eucinostomus_melanopterus	10	omnivore
Caribbean	Gerreidae	Gerres_cinereus	115	omnivore
Caribbean	Gobiidae	Gnatholepis_thompsoni	2	microinvertivore
Caribbean	Muraenidae	Gymnothorax_funebris	3	piscivore
Caribbean	Muraenidae	Gymnothorax_miliaris	3	piscivore
Caribbean	Muraenidae	Gymnothorax_saxicola	1	piscivore
Caribbean	Muraenidae	Gymnothorax_vicinus	6	piscivore
Caribbean	Haemulidae	Haemulon_flavolineatum	18	macroinvertivore
Caribbean	Haemulidae	Haemulon_melanurum	15	macroinvertivore
Caribbean	Haemulidae	Haemulon_parra	7	macroinvertivore
Caribbean	Haemulidae	Haemulon_plumierii	28	macroinvertivore
Caribbean	Haemulidae	Haemulon_sciurus	7	macroinvertivore
Caribbean	Labridae	Halichoeres_bivittatus	19	macroinvertivore
Caribbean	Labridae	Halichoeres_garnoti	3	macroinvertivore
Caribbean	Labridae	Halichoeres_poeyi	2	macroinvertivore
Caribbean	Pomacanthidae	Holacanthus_ciliaris	7	microinvertivore
Caribbean	Holocentridae	Holocentrus_adscensionis	14	macroinvertivore
Caribbean	Holocentridae	Holocentrus_rufus	5	macroinvertivore
Caribbean	Kyphosidae	Kyphosus_sectatrix	2	herbivore.detritivore
Caribbean	Labrisomidae	Labrisomus_nuchipinnis	1	microinvertivore
Caribbean	Labridae	Lachnolaimus_maximus	4	macroinvertivore
Caribbean	Lutjanidae	Lutjanus_analis	2	piscivore.invertivore

Caribbean	Lutjanidae	Lutjanus_apodus	42	piscivore.invertivore
Caribbean	Lutjanidae	Lutjanus_griseus	67	piscivore.invertivore
Caribbean	Lutjanidae	Lutjanus_synagris	14	piscivore.invertivore
Caribbean	Serranidae	Mycteroperca_bonaci	11	piscivore.invertivore
Caribbean	Serranidae	Mycteroperca_venenosa	1	piscivore.invertivore
Caribbean	Labridae	Nicholsina_usta_usta	2	herbivore.detritivore
Caribbean	Lutjanidae	Ocyurus_chrysurus	13	piscivore.invertivore
Caribbean	Sciaenidae	Pareques_acuminatus	6	microinvertivore
Caribbean	Pomacanthidae	Pomacanthus_arcuatus	3	microinvertivore
Caribbean	Pomacanthidae	Pomacanthus_paru	2	microinvertivore
Caribbean	Mullidae	Pseudupeneus_maculatus	3	macroinvertivore
Caribbean	Scorpaenidae	Pterois_volitans	14	piscivore.invertivore
Caribbean	Ostraciidae	Rhinesomus_triqueter	3	macroinvertivore
Caribbean	Blenniidae	Salarias_fasciatus	6	microinvertivore
Caribbean	Holocentridae	Sargocentron_coruscum	5	macroinvertivore
Caribbean	Labridae	Scarus_guacamaia	1	herbivore.detritivore
Caribbean	Labridae	Scarus_iseri	1	herbivore.detritivore
Caribbean	Labridae	Scarus_taeniopterus	1	herbivore.detritivore
Caribbean	Labridae	Sparisoma_chrysopterum	14	herbivore.detritivore
Caribbean	Labridae	Sparisoma_viride	4	herbivore.detritivore
Caribbean	Tetraodontidae	Sphoeroides_testudineus	11	macroinvertivore
Caribbean	Sphyraenidae	Sphyraena_barracuda	2	piscivore
Caribbean	Pomacentridae	Stegastes_adustus	1	herbivore.detritivore
Caribbean	Pomacentridae	Stegastes_leucostictus	17	herbivore.detritivore
Caribbean	Syngnathidae	Syngnathus_fuscus	1	microinvertivore
Caribbean	Syngnathidae	Syngnathus_pelagicus	1	microinvertivore
Caribbean	Labridae	Thalassoma_bifasciatum	6	macroinvertivore
Caribbean	Archiridae	Trinectes_inscriptus	2	macroinvertivore
Caribbean	Belonidae	Tylosurus_crocodilus_crocodilus	9	piscivore
Moorea	Pomacentridae	Abudefduf_septemfasciatus	1	planktivore
Moorea	Pomacentridae	Abudefduf_sexfasciatus	9	planktivore
Moorea	Pomacentridae	Abudefduf_sordidus	1	planktivore
Moorea	Acanthuridae	Acanthurus_nigricans	1	herbivore.detritivore
Moorea	Acanthuridae	Acanthurus_nigrofuscus	6	herbivore.detritivore
Moorea	Acanthuridae	Acanthurus_olivaceus	1	herbivore.detritivore
Moorea	Acanthuridae	Acanthurus_pyroferus	1	herbivore.detritivore
Moorea	Labridae	Anampses_twistii	1	microinvertivore
Moorea	Tetraodontidae	Arothron_meleagris	7	corallivore
Moorea	Aulostomidae	Aulostomus_chinensis	1	piscivore
Moorea	Balistidae	Balistapus_undulatus	39	omnivore

Moorea	Bothidae	Bothus_mancus	1	piscivore.invertivore
Moorea	Bothidae	Bothus_pantherinus	1	piscivore.invertivore
Moorea	Monacanthidae	Cantherhines_sandwichiensis	2	omnivore
Moorea	Tetraodontidae	Canthigaster_solandri	27	omnivore
Moorea	Tetraodontidae	Canthigaster_valentini	2	omnivore
Moorea	Carangidae	Caranx_melampygus	4	piscivore
Moorea	Pomacentridae	Centropyge_bispinosa	1	herbivore.detritivore
Moorea	Pomacentridae	Centropyge_flavissima	5	herbivore.detritivore
Moorea	Serranidae	Cephalopholis_argus	18	piscivore.invertivore
Moorea	Serranidae	Cephalopholis_urodeta	2	piscivore.invertivore
Moorea	Chaetodontidae	Chaetodon_citrinellus	33	corallivore
Moorea	Chaetodontidae	Chaetodon_ephippium	1	corallivore
Moorea	Chaetodontidae	Chaetodon_lunula	6	corallivore
Moorea	Chaetodontidae	Chaetodon_lunulatus	17	corallivore
Moorea	Chaetodontidae	Chaetodon_ornatissimus	11	corallivore
Moorea	Chaetodontidae	Chaetodon_quadrimaculatus	7	corallivore
Moorea	Chaetodontidae	Chaetodon_reticulatus	5	corallivore
Moorea	Chaetodontidae	Chaetodon_ulietensis	1	corallivore
Moorea	Chaetodontidae	Chaetodon_vagabundus	7	corallivore
Moorea	Labridae	Cheilinus_chlorourus	3	microinvertivore
Moorea	Labridae	Cheilinus_trilobatus	1	microinvertivore
Moorea	Labridae	Cheilio_inermis	2	microinvertivore
Moorea	Apogonidae	Cheilodipterus_quinquelineatus	1	piscivore.invertivore
Moorea	Pomacentridae	Chromis_atripectoralis	3	planktivore
Moorea	Pomacentridae	Chromis_margaritifer	2	planktivore
Moorea	Pomacentridae	Chromis_viridis	11	planktivore
Moorea	Pomacentridae	Chrysiptera_brownriggii	3	herbivore.detritivore
Moorea	Labridae	Cirrhilabrus_scottorum	2	microinvertivore
Moorea	Labridae	Coris_aygula	1	microinvertivore
Moorea	Labridae	Coris_gaimard	2	microinvertivore
Moorea	Mugilidae	Crenimugil_crenilabis	8	herbivore.detritivore
Moorea	Acanthuridae	Ctenochaetus_striatus	44	herbivore.detritivore
Moorea	Pomacentridae	Dascyllus_aruanus	12	planktivore
Moorea	Pomacentridae	Dascyllus_flavicaudus	14	planktivore
Moorea	Pomacentridae	Dascyllus_trimaculatus	45	planktivore
Moorea	Diodontidae	Diodon_hystrix	3	macroinvertivore
Moorea	Labridae	Epibulus_insidiator	4	piscivore.invertivore
Moorea	Serranidae	Epinephelus_merra	51	piscivore.invertivore
Moorea	Fistulariidae	Fistularia_commersonii	4	piscivore
Moorea	Chaetodontidae	Forcipiger_flavissimus	14	microinvertivore

Moorea	Chaetodontidae	Forcipiger_longirostris	1	microinvertivore
Moorea	Lethrinidae	Gnathodentex_aureolineatus	20	macroinvertivore
Moorea	Labridae	Gomphosus_varius	8	microinvertivore
Moorea	Labridae	Halichoeres_hortulanus	16	microinvertivore
Moorea	Labridae	Halichoeres_marginatus	6	microinvertivore
Moorea	Labridae	Halichoeres_trimaculatus	22	microinvertivore
Moorea	Chaetodontidae	Heniochus_chrysostomus	4	corallivore
Moorea	Blenniidae	Istiblennius_edentulus	1	herbivore.detritivore
Moorea	Labridae	Labroides_bicolor	3	microinvertivore
Moorea	Lutjanidae	Lutjanus_fulvus	12	piscivore.invertivore
Moorea	Lutjanidae	Lutjanus_kasmira	1	macroinvertivore
Moorea	Balistidae	Melichthys_niger	2	omnivore
Moorea	Balistidae	Melichthys_vidua	8	omnivore
Moorea	Lethrinidae	Monotaxis_grandoculis	7	macroinvertivore
Moorea	Mullidae	Mulloidichthys_vanicolensis	7	macroinvertivore
Moorea	Holocentridae	Myripristis_amaena	9	planktivore
Moorea	Holocentridae	Myripristis_berndti	1	planktivore
Moorea	Holocentridae	Myripristis_kuntee	7	planktivore
Moorea	Acanthuridae	Naso_lituratus	1	herbivore.detritivore
Moorea	Holocentridae	Neoniphon_argenteus	9	macroinvertivore
Moorea	Holocentridae	Neoniphon_sammara	13	macroinvertivore
Moorea	Balistidae	Odonus_niger	1	planktivore
Moorea	Ostraciidae	Ostracion_meleagris	4	microinvertivore
Moorea	Labridae	Oxycheilinus_unifasciatus	2	piscivore.invertivore
Moorea	Cirrhitidae	Paracirrhites_arcatus	2	microinvertivore
Moorea	Pinguipedidae	Parapercis_millepunctata	3	macroinvertivore
Moorea	Mullidae	Parupeneus_ciliatus	3	macroinvertivore
Moorea	Mullidae	Parupeneus_cyclostomus	1	macroinvertivore
Moorea	Mullidae	Parupeneus_insularis	2	macroinvertivore
Moorea	Mullidae	Parupeneus_multifasciatus	13	macroinvertivore
Moorea	Pomacentridae	Pomacentrus_pavo	38	planktivore
Moorea	Pomacentridae	Pomachromis_fuscidorsalis	3	planktivore
Moorea	Apogonidae	Pristiapogon_exostigma	2	piscivore.invertivore
Moorea	Apogonidae	Pristiapogon_kallopterus	7	planktivore
Moorea	Labridae	Pseudocheilinus_hexataenia	1	microinvertivore
Moorea	Labridae	Pseudojuloides_atavai	1	microinvertivore
Moorea	Scorpaenidae	Pterois_antennata	2	piscivore.invertivore
Moorea	Pomacentridae	Pygoplites_diacanthus	1	microinvertivore
Moorea	Balistidae	Rhinecanthus_aculeatus	4	omnivore
Moorea	Holocentridae	Sargocentron_caudimaculatum	35	macroinvertivore

Moorea	Holocentridae	Sargocentron_diadema	3	macroinvertivore
Moorea	Holocentridae	Sargocentron_microstoma	2	macroinvertivore
Moorea	Holocentridae	Sargocentron_punctatissimum	1	macroinvertivore
Moorea	Holocentridae	Sargocentron_spiniferum	1	macroinvertivore
Moorea	Labridae	Scarus_globiceps	1	herbivore.detritivore
Moorea	Labridae	Scarus_oviceps	3	herbivore.detritivore
Moorea	Labridae	Scarus_psittacus	18	herbivore.detritivore
Moorea	Pomacentridae	Stegastes_fasciolatus	6	herbivore.detritivore
Moorea	Pomacentridae	Stegastes_nigricans	3	herbivore.detritivore
Moorea	Labridae	Stethojulis_bandanensis	9	microinvertivore
Moorea	Balistidae	Sufflamen_bursa	27	omnivore
Moorea	Syondontidae	Synodus_dermatogenys	1	piscivore.invertivore
Moorea	Syondontidae	Synodus_jaculum	1	piscivore.invertivore
Moorea	Labridae	Thalassoma_hardwicke	17	microinvertivore
Moorea	Syondontidae	Valenciennea_strigata	3	microinvertivore
Moorea	Zanclidae	Zanclus_cornutus	5	microinvertivore
Moorea	Acanthuridae	Zebrasoma_scopas	38	herbivore.detritivore

Figure 4

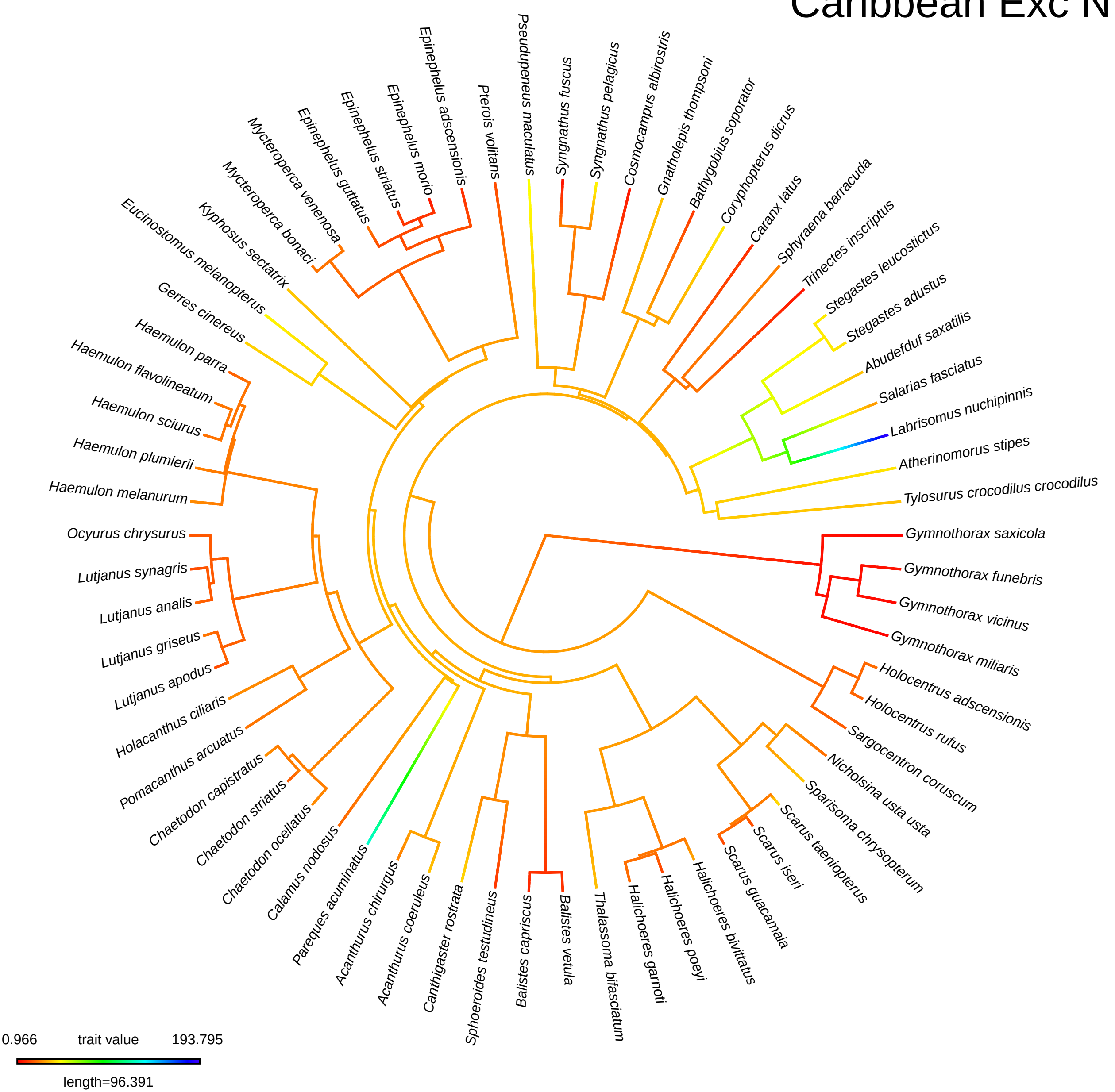

Caribbean Exc N
Pseudupeneus maculatus
Syngnathus fuscus
Syngnathus pelagicus
Cosmocampus albirostris
Gnatholepis thompsoni
Bathygobius soporator
Coryphopterus dicrus
Caranx latus
Sphyraena barracuda
Trinectes inscriptus
Stegastes leucostictus
Stegastes adustus
Abudefduf saxatilis
Salarias fasciatus
Labrisomus nuchipinnis
Atherinomorus stipes
Tylosurus crocodilus crocodilus
Gymnothorax saxicola
Gymnothorax funebris
Gymnothorax vicinus
Gymnothorax miliaris
Holocentrus adscensionis
Holocentrus rufus
Sargocentron coruscum
Nicholsina usta usta
Sparisoma chrysopterum
Scarus taeniopterus
Scarus iseri
Scarus guacamaia
Halichoeres bivittatus
Halichoeres poeyi
Halichoeres garnoti
Thalassoma bifasciatum
Balistes vetula
Balistes capriscus
Sphoeroides testudineus
Canthigaster rostrata
Acanthurus coeruleus
Acanthurus chirurgus
Pareques acuminatus
Calamus nodosus
Chaetodon ocellatus
Chaetodon striatus
Chaetodon capistratus
Pomacanthus arcuatus
Holacanthus ciliaris
Lutjanus apodus
Lutjanus griseus
Lutjanus analis
Lutjanus synagris
Ocyurus chrysurus
Haemulon melanurum
Haemulon plumierii
Haemulon sciurus
Haemulon flavolineatum
Haemulon parra
Gerres cinereus
Eucinostomus melanopterus
Kyphosus sectatrix
Mycteroperca bonaci
Mycteroperca venenosa
Epinephelus guttatus
Epinephelus striatus
Epinephelus morio
Epinephelus adscensionis
Pterois volitans
0.966
trait value
193.795
length=96.391

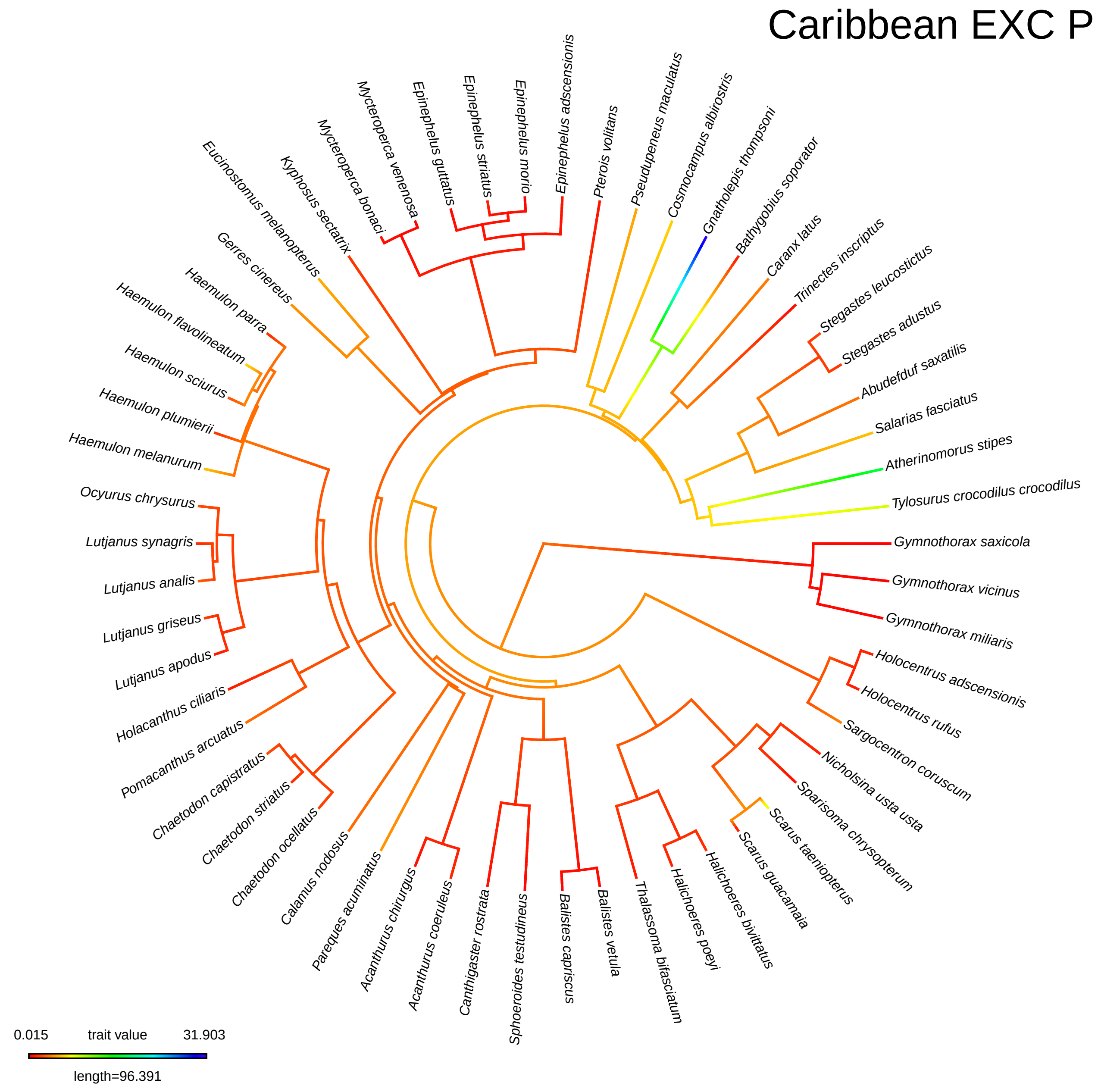

Caribbean EXC P
Epinephelus adscensionis
Epinephelus morio
Epinephelus striatus
Epinephelus guttatus
Mycteroperca venenosa
Mycteroperca bonaci
Kyphosus sectatrix
Eucinostomus melanopterus
Gerres cinereus
Haemulon parra
Haemulon flavolineatum
Haemulon sciurus
Haemulon plumierii
Haemulon melanurum
Ocyurus chrysurus
Lutjanus synagris
Lutjanus analis
Lutjanus griseus
Lutjanus apodus
Holacanthus ciliaris
Pomacanthus arcuatus
Chaetodon capistratus
Chaetodon striatus
Chaetodon ocellatus
Calamus nodosus
Pareques acuminatus
Acanthurus chirurgus
Acanthurus coeruleus
Canthigaster rostrata
Sphoeroides testudineus
Balistes capriscus
Balistes vetula
Thalassoma bifasciatum
Halichoeres poeyi
Halichoeres bivittatus
Scarus guacamaia
Scarus taeniopterus
Sparisoma chrysopterum
Nicholsina usta usta
Sargocentron coruscum
Holocentrus rufus
Holocentrus adscensionis
Gymnothorax miliaris
Gymnothorax vicinus
Gymnothorax saxicola
Tylosurus crocodilus crocodilus
Atherinomorus stipes
Salarias fasciatus
Abudefduf saxatilis
Stegastes adustus
Stegastes leucostictus
Trinectes inscriptus
Caranx latus
Bathygobius soporator
Gnatholepis thompsoni
Cosmocampus albirostris
Pseudupeneus maculatus
Pterois volitans
0.015
trait value
31.903
length=96.391

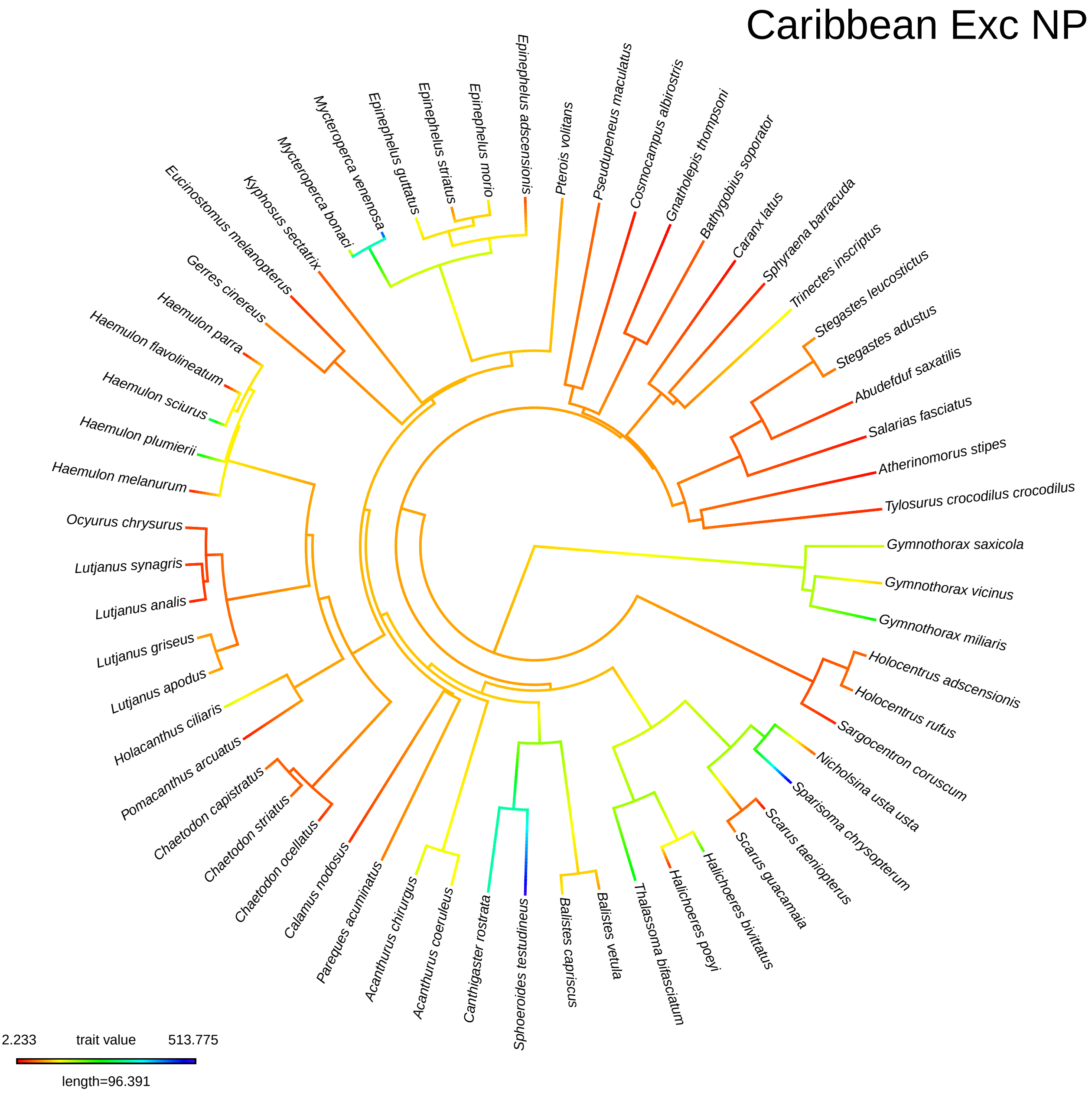

Caribbean Exc NP
Epinephelus adscensionis
Pterois volitans
Pseudupeneus maculatus
Cosmocampus albirostris
Gnatholepis thompsoni
Bathygobius soporator
Caranx latus
Sphyraena barracuda
Trinectes inscriptus
Stegastes leucostictus
Stegastes adustus
Abudefduf saxatilis
Salarias fasciatus
Atherinomorus stipes
Tylosurus crocodilus crocodilus
Gymnothorax saxicola
Gymnothorax vicinus
Gymnothorax miliaris
Holocentrus adscensionis
Holocentrus rufus
Sargocentron coruscum
Nicholsina usta usta
Sparisoma chrysopterum
Scarus taeniopterus
Scarus guacamaia
Halichoeres bivittatus
Halichoeres poeyi
Thalassoma bifasciatum
Balistes vetula
Balistes capriscus
Sphoeroides testudineus
Canthigaster rostrata
Acanthurus coeruleus
Acanthurus chirurgus
Pareques acuminatus
Calamus nodosus
Chaetodon ocellatus
Chaetodon striatus
Chaetodon capistratus
Pomacanthus arcuatus
Holacanthus ciliaris
Lutjanus apodus
Lutjanus griseus
Lutjanus analis
Lutjanus synagris
Ocyurus chrysurus
Haemulon melanurum
Haemulon plumierii
Haemulon sciurus
Haemulon flavolineatum
Haemulon parra
Gerres cinereus
Eucinostomus melanopterus
Kyphosus sectatrix
Mycteroperca bonaci
Mycteroperca venenosa
Epinephelus guttatus
Epinephelus striatus
Epinephelus morio
2.233
trait value
513.775
length=96.391

Caribbean Body C

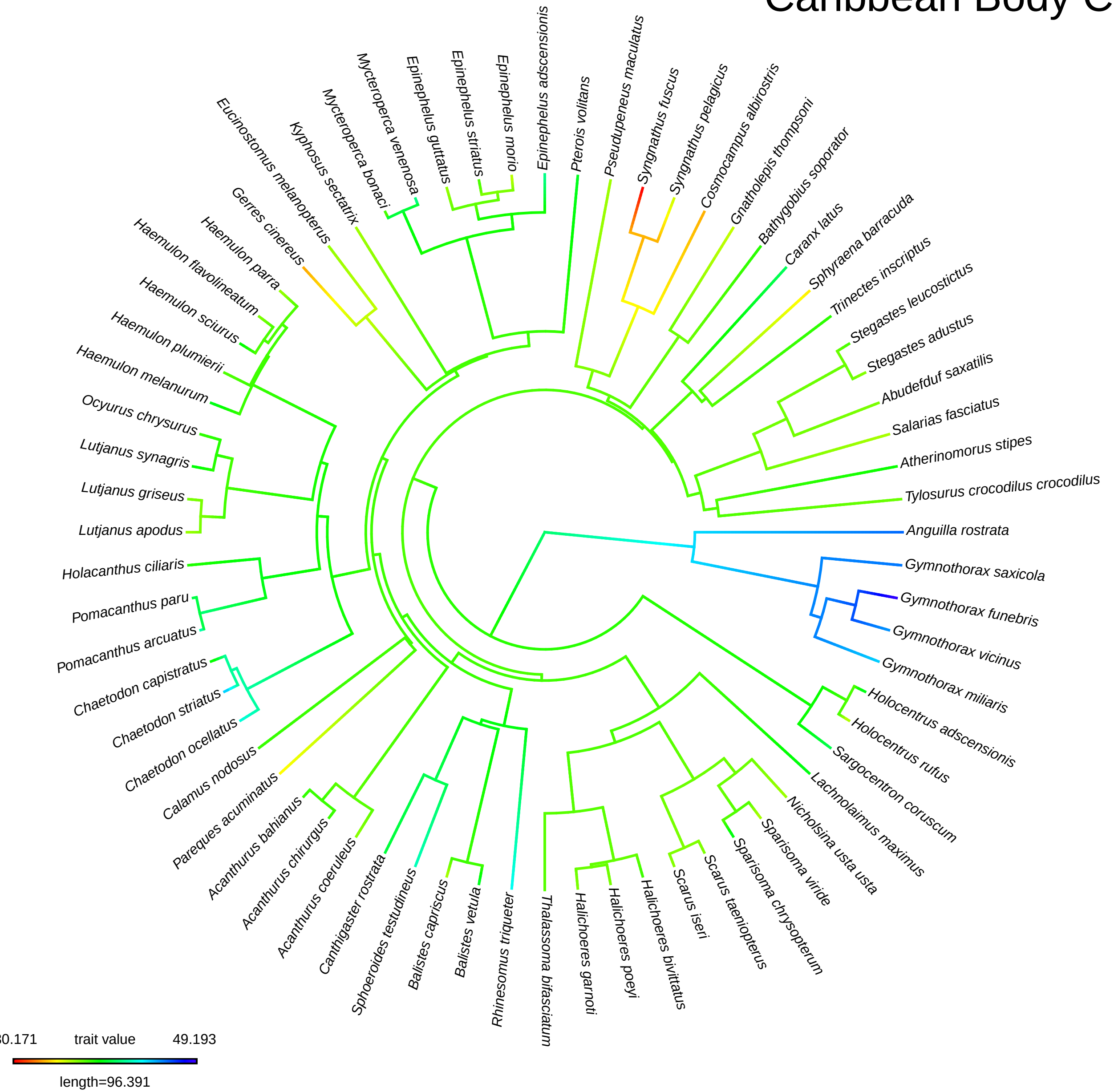

Caribbean Body N

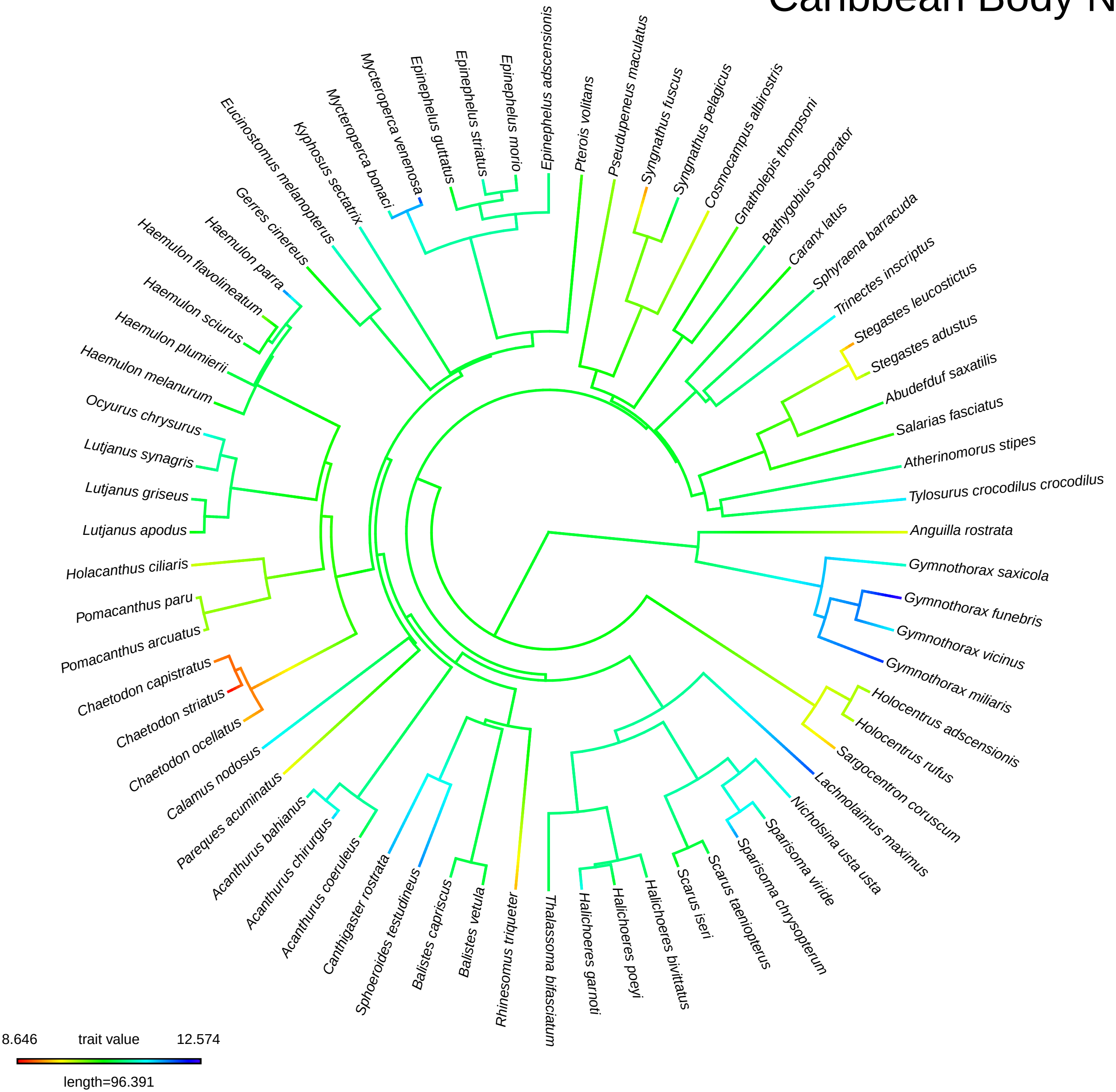

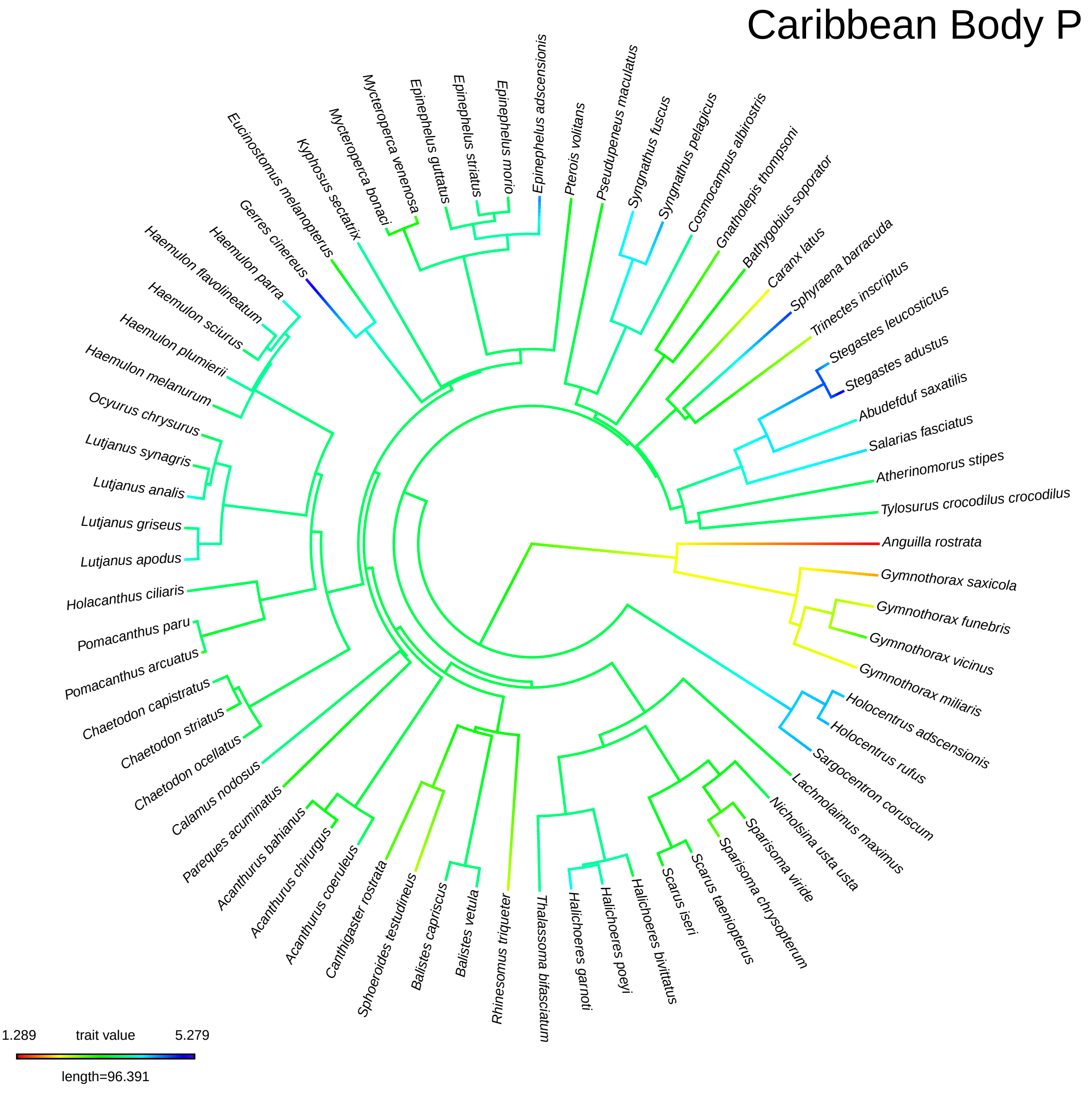

Caribbean Body P
Eucinostomus melanopterus
Kyphosus sectatrix
Mycteroperca bonaci
Mycteroperca venenosa
Epinephelus guttatus
Epinephelus striatus
Epinephelus morio
Epinephelus adscensionis
Pterois volitans
Pseudupeneus maculatus
Syngnathus fuscus
Syngnathus pelagicus
Cosmocampus albirostris
Gnatholepis thompsoni
Bathygobius soporator
Caranx latus
Sphyraena barracuda
Trinectes inscriptus
Stegastes leucostictus
Stegastes adustus
Abudefduf saxatilis
Salarias fasciatus
Atherinomorus stipes
Tylosurus crocodilus crocodilus
Anguilla rostrata
Gymnothorax saxicola
Gymnothorax funebris
Gymnothorax vicinus
Gymnothorax miliaris
Holocentrus adscensionis
Holocentrus rufus
Sargocentron coruscum
Lachnolaimus maximus
Nicholsina usta usta
Sparisoma viride
Sparisoma chrysopterum
Scarus taeniopterus
Scarus iseri
Halichoeres bivittatus
Halichoeres poeyi
Halichoeres garnoti
Thalassoma bifasciatum
Rhinesomus triqueter
Balistes vetula
Balistes capriscus
Sphoeroides testudineus
Canthigaster rostrata
Acanthurus coeruleus
Acanthurus chirurgus
Acanthurus bahianus
Pareques acuminatus
Calamus nodosus
Chaetodon ocellatus
Chaetodon striatus
Chaetodon capistratus
Pomacanthus arcuatus
Pomacanthus paru
Holacanthus ciliaris
Lutjanus apodus
Lutjanus griseus
Lutjanus analis
Lutjanus synagris
Ocyurus chrysurus
Haemulon melanurum
Haemulon plumierii
Haemulon sciurus
Haemulon flavolineatum
Haemulon parra
Gerres cinereus
1.289
trait value
5.279
length=96.391

Caribbean Body CN

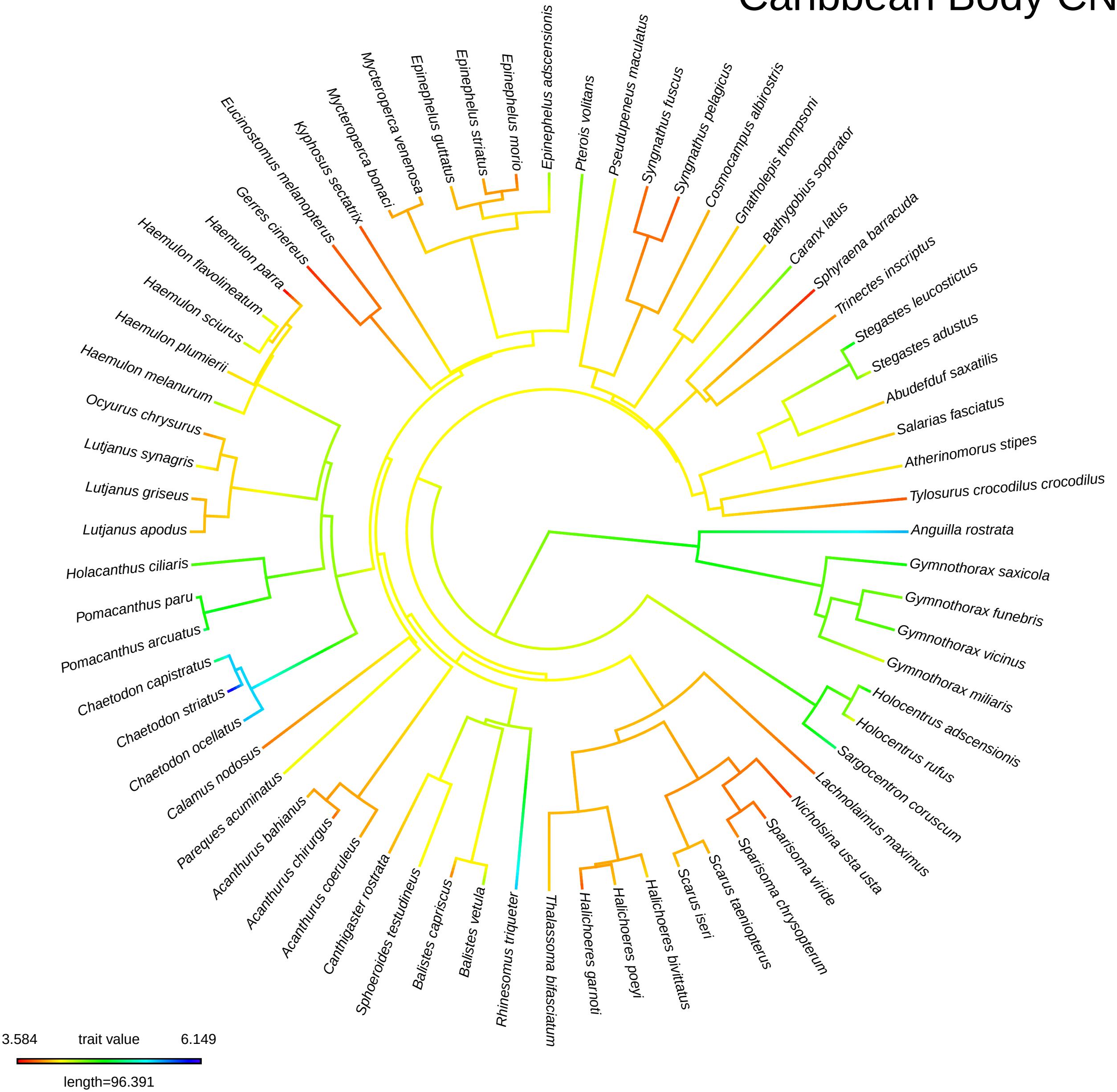

Caribbean Body CP

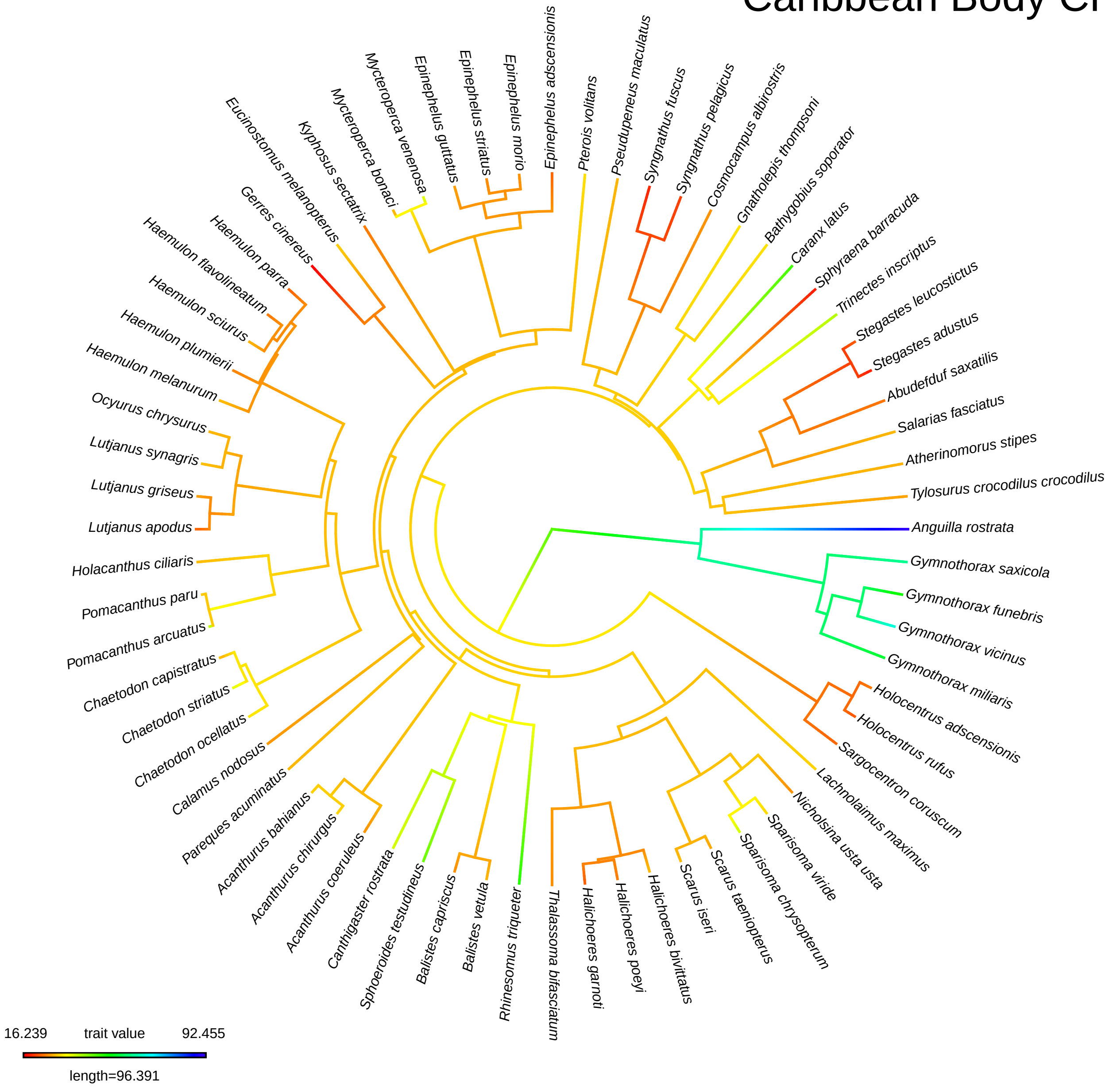

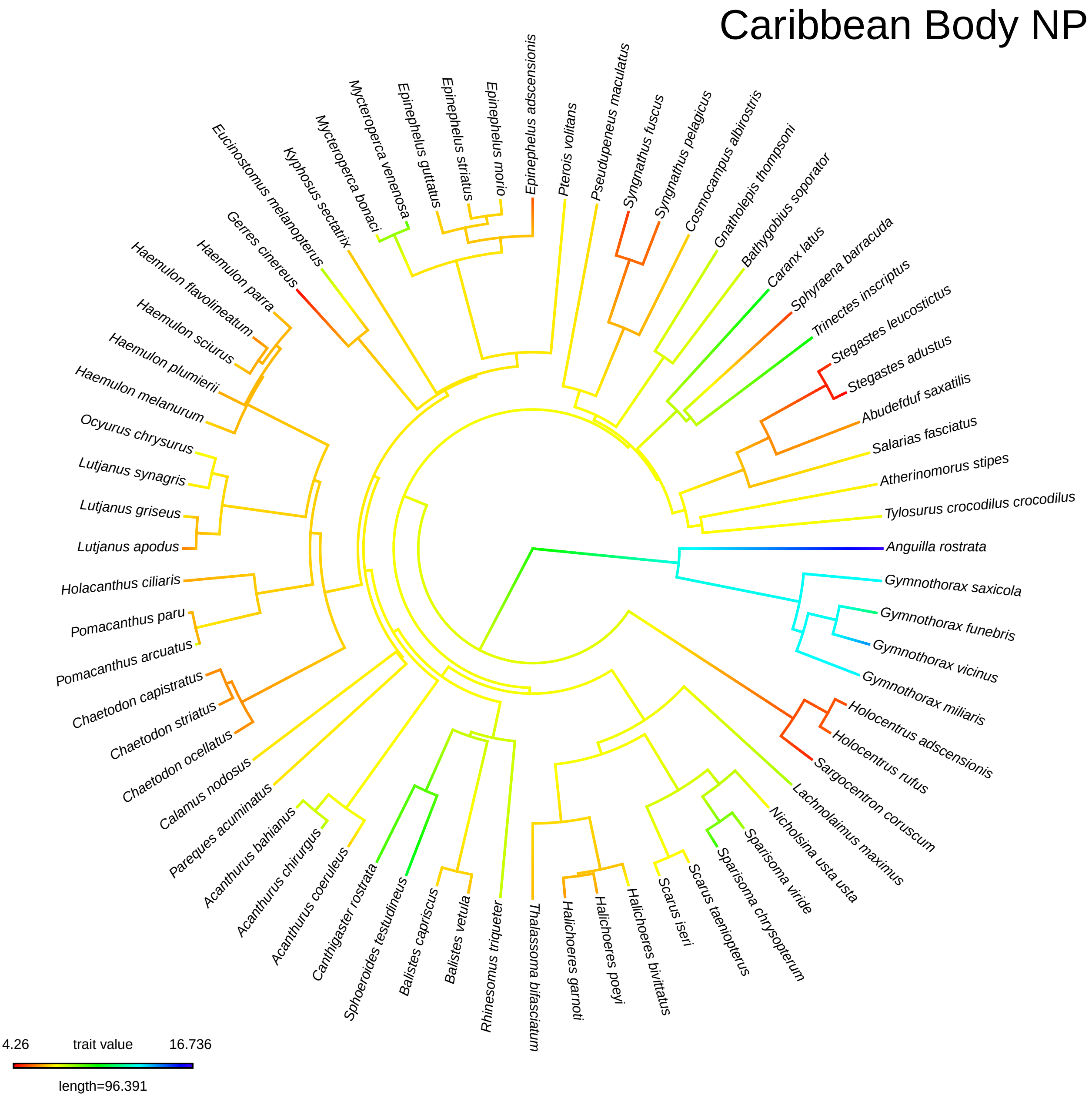
Caribbean Body NP
Epinephelus adscensionis
Pterois volitans
Pseudupeneus maculatus
Syngnathus fuscus
Syngnathus pelagicus
Cosmocampus albirostris
Gnatholepis thompsoni
Bathygobius soporator
Caranx latus
Sphyraena barracuda
Trinectes inscriptus
Stegastes leucostictus
Stegastes adustus
Abudefduf saxatilis
Salarias fasciatus
Atherinomorus stipes
Tylosurus crocodilus crocodilus
Anguilla rostrata
Gymnothorax saxicola
Gymnothorax funebris
Gymnothorax vicinus
Gymnothorax miliaris
Holocentrus adscensionis
Holocentrus rufus
Sargocentron coruscum
Lachnolaimus maximus
Nicholsina usta usta
Sparisoma viride
Sparisoma chrysopterum
Scarus taeniopterus
Scarus iseri
Halichoeres bivittatus
Halichoeres poeyi
Halichoeres garnoti
Thalassoma bifasciatum
Rhinesomus triqueter
Balistes vetula
Balistes capriscus
Sphoeroides testudineus
Canthigaster rostrata
Acanthurus coeruleus
Acanthurus chirurgus
Acanthurus bahianus
Pareques acuminatus
Calamus nodosus
Chaetodon ocellatus
Chaetodon striatus
Chaetodon capistratus
Pomacanthus arcuatus
Pomacanthus paru
Holacanthus ciliaris
Lutjanus apodus
Lutjanus griseus
Lutjanus synagris
Ocyurus chrysurus
Haemulon melanurum
Haemulon plumierii
Haemulon sciurus
Haemulon flavolineatum
Haemulon parra
Gerres cinereus
Eucinostomus melanopterus
Kyphosus sectatrix
Mycteroperca bonaci
Mycteroperca venenosa
Epinephelus guttatus
Epinephelus striatus
Epinephelus morio
4.26
trait value
16.736
length=96.391

Figure 5

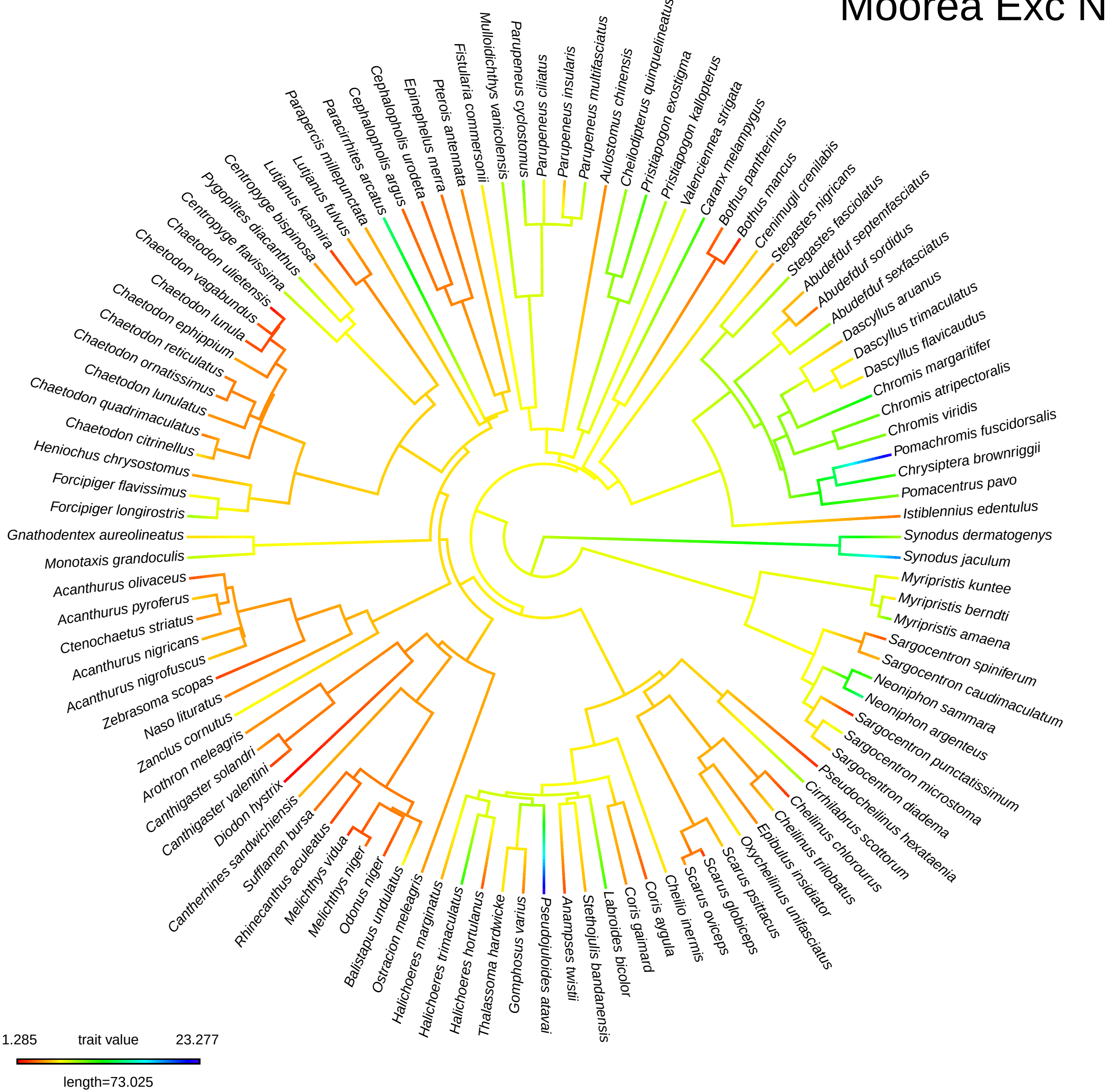
Moorea Exc N
Fistularia commersonii
Mulloidichthys vanicolensis
Parupeneus cyclostomus
Parupeneus ciliatus
Parupeneus insularis
Parupeneus multifasciatus
Aulostomus chinensis
Cheilodipterus quinquelineatus
Pristiapogon exostigma
Pristiapogon kallopterus
Valenciennea strigata
Caranx melampygus
Bothus pantherinus
Bothus mancus
Crenimugil crenilabis
Stegastes nigricans
Stegastes fasciolatus
Abudefduf septemfasciatus
Abudefduf sordidus
Abudefduf sexfasciatus
Dascyllus aruanus
Dascyllus trimaculatus
Dascyllus flavicaudus
Chromis margaritifer
Chromis atripectoralis
Chromis viridis
Pomachromis fuscidorsalis
Chrysiptera brownriggii
Pomacentrus pavo
Istiblennius edentulus
Synodus dermatogenys
Synodus jaculum
Myripristis kuntee
Myripristis berndti
Myripristis amaena
Sargocentron spiniferum
Sargocentron caudimaculatum
Neoniphon sammara
Neoniphon argenteus
Sargocentron punctatissimum
Sargocentron microstoma
Sargocentron diadema
Pseudocheilinus hexataenia
Cirrhilabrus scottorum
Cheilinus chlorourus
Cheilinus trilobatus
Epibulus insidiator
Oxycheilinus unifasciatus
Scarus psittacus
Scarus globiceps
Scarus oviceps
Chelio inermis
Coris aygula
Coris gaimard
Labroides bicolor
Stethojulis bandanensis
Anampses twistii
Pseudojuloides atavai
Gomphosus varius
Thalassoma hardwicke
Halichoeres hortulanus
Halichoeres trimaculatus
Halichoeres marginatus
Ostracion meleagris
Balistapus undulatus
Odonus niger
Melichthys niger
Melichthys vidua
Rhinecanthus aculeatus
Sufflamen bursa
Cantherhines sandwichiensis
Diodon hystrix
Canthigaster valentini
Canthigaster solandri
Arothron meleagris
Zanclus cornutus
Naso lituratus
Zebrasoma scopas
Acanthurus nigrofuscus
Acanthurus nigricans
Ctenochaetus striatus
Acanthurus pyroferus
Acanthurus olivaceus
Monotaxis grandoculis
Gnathodentex aureolineatus
Forcipiger longirostris
Forcipiger flavissimus
Heniochus chrysostomus
Chaetodon citrinellus
Chaetodon quadrimaculatus
Chaetodon lunulatus
Chaetodon ornatissimus
Chaetodon reticulatus
Chaetodon ephippium
Chaetodon lunula
Chaetodon vagabundus
Chaetodon ulietensis
Centropyge flavissima
Pygoplites diacanthus
Centropyge bispinosa
Lutjanus kasmira
Lutjanus fulvus
Parapercis millepunctata
Paracirrhites arcatus
Cephalopholis argus
Cephalopholis urodeta
Epinephelus merra
Pterois antennata
1.285
trait value
23.277
length=73.025

Moorea EXC P

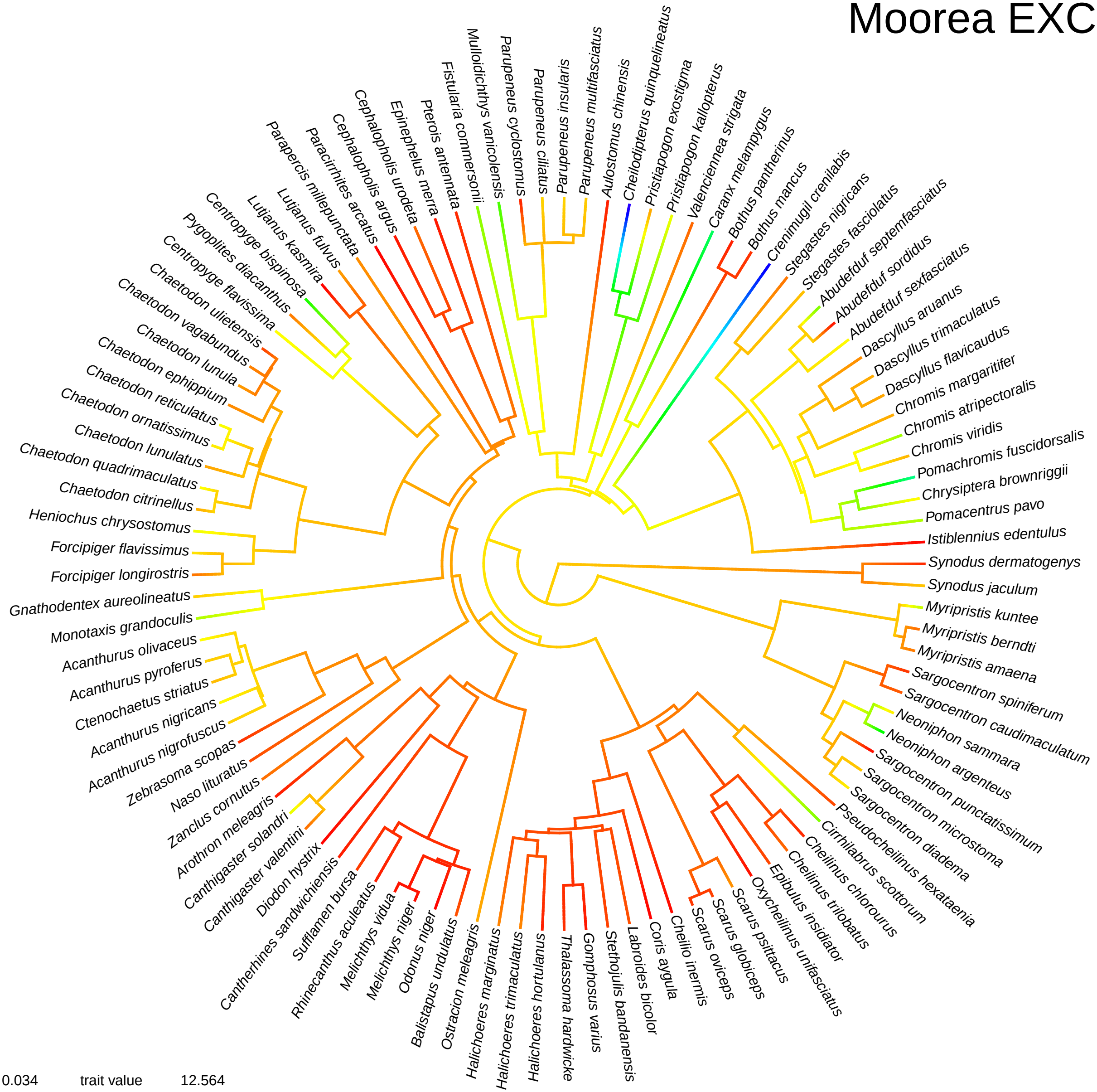

0.034 trait value 12.564

length=73.025

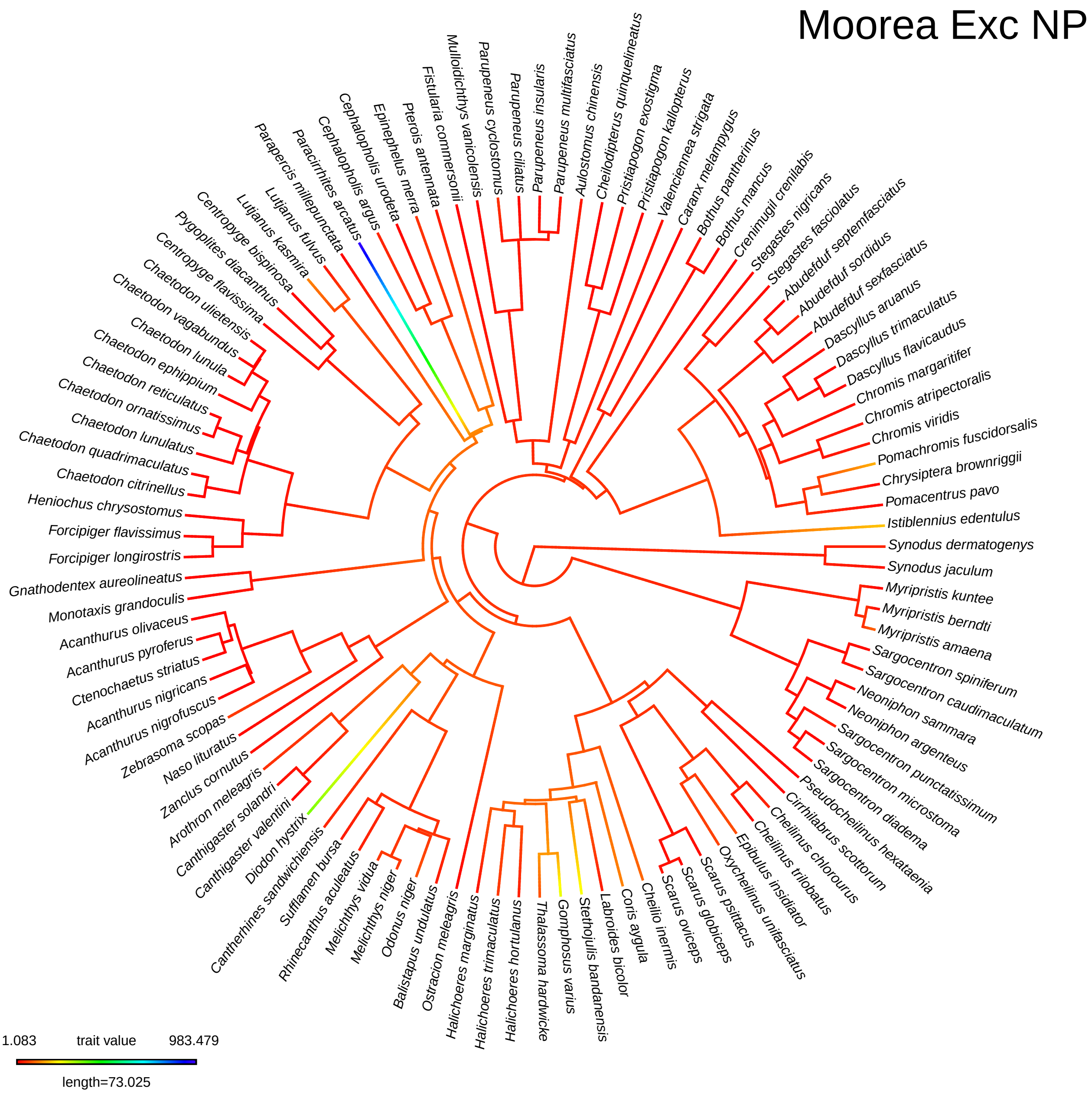
Moorea Exc NP
Synodus dermatogenys
Synodus jaculum
Myripristis kuntee
Myripristis berndti
Myripristis amaena
Sargocentron spiniferum
Sargocentron caudimaculatum
Neoniphon sammara
Neoniphon argenteus
Sargocentron punctatissimum
Sargocentron microstoma
Sargocentron diadema
Pseudocheilinus hexataenia
Cirrhilabrus scottorum
Cheilinus chlorourus
Cheilinus trilobatus
Epibulus insidiator
Oxycheilinus unifasciatus
Scarus psittacus
Scarus globiceps
Scarus oviceps
Cheilio inermis
Coris aygula
Labroides bicolor
Stethojulis bandanensis
Gomphosus varius
Thalassoma hardwicke
Halichoeres hortulanus
Halichoeres trimaculatus
Halichoeres marginatus
Ostracion meleagris
Balistapus undulatus
Odonus niger
Melichthys niger
Melichthys vidua
Rhinecanthus aculeatus
Sufflamen bursa
Cantherhines sandwichiensis
Diodon hystrix
Canthigaster valentini
Canthigaster solandri
Arothron meleagris
Zanclus cornutus
Naso lituratus
Zebrasoma scopas
Acanthurus nigrofuscus
Acanthurus nigricans
Ctenochaetus striatus
Acanthurus pyroferus
Acanthurus olivaceus
Monotaxis grandoculis
Gnathodentex aureolineatus
Forcipiger longirostris
Forcipiger flavissimus
Heniochus chrysostomus
Chaetodon citrinellus
Chaetodon quadrimaculatus
Chaetodon lunulatus
Chaetodon ornatissimus
Chaetodon reticulatus
Chaetodon ephippium
Chaetodon lunula
Chaetodon vagabundus
Chaetodon ulietensis
Centropyge flavissima
Pygoplites diacanthus
Centropyge bispinosa
Lutjanus kasmira
Lutjanus fulvus
Parapercis millepunctata
Paracirrhites arcatus
Cephalopholis argus
Cephalopholis urodeta
Epinephelus merra
Pterois antennata
Fistularia commersonii
Mulloidichthys vanicolensis
Parupeneus cyclostomus
Parupeneus ciliatus
Parupeneus insularis
Parupeneus multifasciatus
Aulostomus chinensis
Cheilodipterus quinquelineatus
Pristiapogon exostigma
Pristiapogon kallopterus
Valenciennea strigata
Caranx melampygus
Bothus pantherinus
Bothus mancus
Crenimugil crenilabis
Stegastes nigricans
Stegastes fasciolatus
Abudefduf septemfasciatus
Abudefduf sordidus
Abudefduf sexfasciatus
Dascyllus aruanus
Dascyllus trimaculatus
Dascyllus flavicaudus
Chromis margaritifer
Chromis atripectoralis
Chromis viridis
Pomachromis fuscidorsalis
Chrysiptera brownriggii
Pomacentrus pavo
Istiblennius edentulus
1.083
trait value
983.479
length=73.025

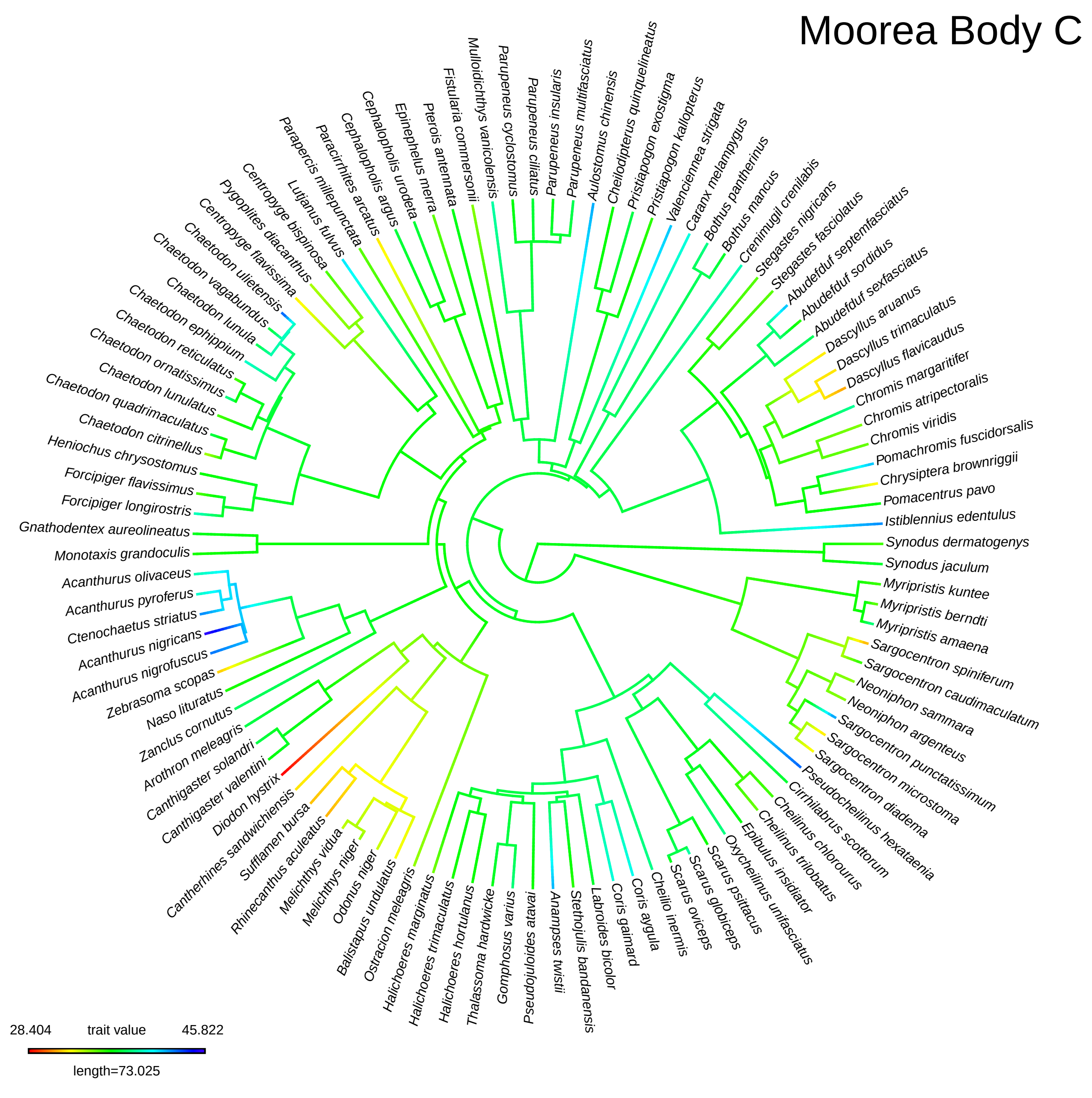
Moorea Body C
Gnathodentex aureolineatus
Monotaxis grandoculis
Forcipiger longirostris
Forcipiger flavissimus
Heniochus chrysostomus
Chaetodon citrinellus
Chaetodon quadrimaculatus
Chaetodon lunulatus
Chaetodon ornatissimus
Chaetodon reticulatus
Chaetodon ephippium
Chaetodon lunula
Chaetodon vagabundus
Chaetodon ulietensis
Centropyge flavissima
Pygoplites diacanthus
Centropyge bispinosa
Lutjanus fulvus
Parapercis millepunctata
Paracirrhites arcatus
Cephalopholis argus
Cephalopholis urodeta
Epinephelus merra
Pterois antennata
Fistularia commersonii
Mulloidichthys vanicolensis
Parupeneus cyclostomus
Parupeneus ciliatus
Parupeneus insularis
Parupeneus multifasciatus
Aulostomus chinensis
Cheilodipterus quinquelineatus
Pristiapogon exostigma
Pristiapogon kallopterus
Valenciennea strigata
Caranx melampygus
Bothus pantherinus
Bothus mancus
Crenimugil crenilabis
Stegastes nigricans
Stegastes fasciolatus
Abudefduf septemfasciatus
Abudefduf sordidus
Abudefduf sexfasciatus
Dascyllus aruanus
Dascyllus trimaculatus
Dascyllus flavicaudus
Chromis margaritifer
Chromis atripectoralis
Chromis viridis
Pomachromis fuscidorsalis
Chrysiptera brownriggii
Pomacentrus pavo
Istiblennius edentulus
Synodus dermatogenys
Synodus jaculum
Myripristis kuntee
Myripristis berndti
Myripristis amaena
Sargocentron spiniferum
Sargocentron caudimaculatum
Neoniphon sammara
Neoniphon argenteus
Sargocentron punctatissimum
Sargocentron microstoma
Sargocentron diadema
Pseudocheilinus hexataenia
Cirrhilabrus scottorum
Cheilinus chlorourus
Cheilinus trilobatus
Epibulus insidiator
Oxycheilinus unifasciatus
Scarus psittacus
Scarus globiceps
Scarus oviceps
Cheilio inermis
Coris aygula
Coris gaimard
Labroides bicolor
Stethojulis bandanensis
Anampses twistii
Pseudojuloides atavai
Gomphosus varius
Thalassoma hardwicke
Halichoeres hortulanus
Halichoeres trimaculatus
Halichoeres marginatus
Ostracion meleagris
Balistapus undulatus
Odonus niger
Melichthys niger
Melichthys vidua
Rhinecanthus aculeatus
Sufflamen bursa
Cantherhines sandwichiensis
Diodon hystrix
Canthigaster valentini
Canthigaster solandri
Arothron meleagris
Zanclus cornutus
Naso lituratus
Zebrasoma scopas
Acanthurus nigrofuscus
Acanthurus nigricans
Ctenochaetus striatus
Acanthurus pyroferus
Acanthurus olivaceus
28.404
trait value
45.822
length=73.025

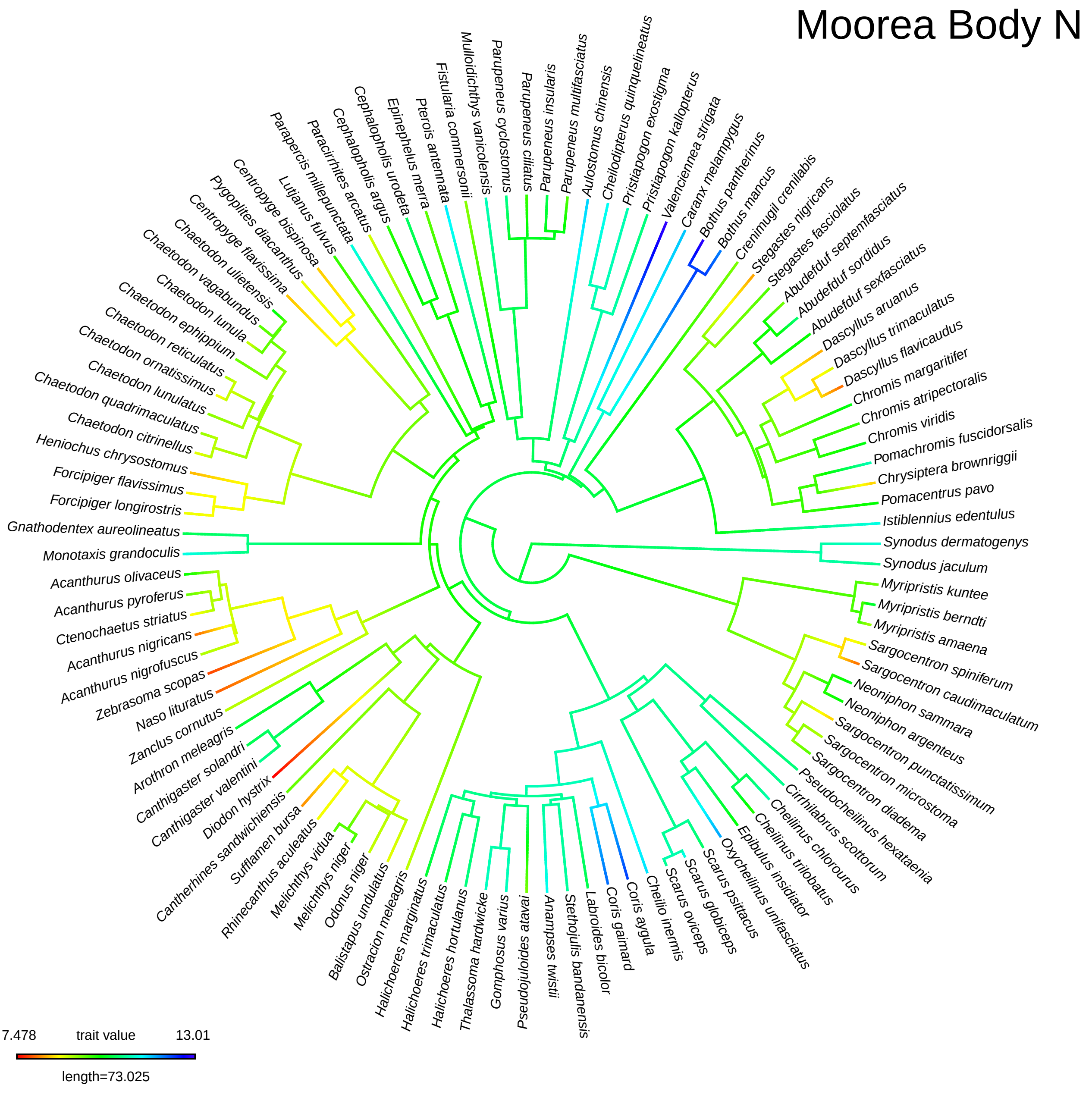
Moorea Body N
Synodus dermatogenys
Synodus jaculum
Myripristis kuntee
Myripristis berndti
Myripristis amaena
Sargocentron spiniferum
Sargocentron caudimaculatum
Neoniphon sammara
Neoniphon argenteus
Sargocentron punctatissimum
Sargocentron microstoma
Sargocentron diadema
Pseudocheilinus hexataenia
Cirrhilabrus scottorum
Cheilinus chlorourus
Cheilinus trilobatus
Epibulus insidiator
Oxycheilinus unifasciatus
Scarus psittacus
Scarus globiceps
Scarus oviceps
Cheilio inermis
Coris aygula
Coris gaimard
Labroides bicolor
Stethojulis bandanensis
Anampses twistii
Pseudojuloides atavai
Gomphosus varius
Thalassoma hardwicke
Halichoeres hortulanus
Halichoeres trimaculatus
Halichoeres marginatus
Ostracion meleagris
Balistapus undulatus
Odonus niger
Melichthys niger
Melichthys vidua
Rhinecanthus aculeatus
Sufflamen bursa
Cantherhines sandwichiensis
Diodon hystrix
Canthigaster valentini
Canthigaster solandri
Arothron meleagris
Zanclus cornutus
Naso lituratus
Zebrasoma scopas
Acanthurus nigrofuscus
Acanthurus nigricans
Ctenochaetus striatus
Acanthurus pyroferus
Acanthurus olivaceus
Monotaxis grandoculis
Gnathodentex aureolineatus
Forcipiger longirostris
Forcipiger flavissimus
Heniochus chrysostomus
Chaetodon citrinellus
Chaetodon quadrimaculatus
Chaetodon lunulatus
Chaetodon ornatissimus
Chaetodon reticulatus
Chaetodon ephippium
Chaetodon lunula
Chaetodon vagabundus
Chaetodon ulietensis
Centropyge flavissima
Pygoplites diacanthus
Centropyge bispinosa
Lutjanus fulvus
Parapercis millepunctata
Paracirrhites arcatus
Cephalopholis argus
Cephalopholis urodeta
Epinephelus merra
Pterois antennata
Fistularia commersonii
Mulloidichthys vanicolensis
Parupeneus cyclostomus
Parupeneus ciliatus
Parupeneus insularis
Parupeneus multifasciatus
Aulostomus chinensis
Cheilodipterus quinquelineatus
Pristiapogon exostigma
Pristiapogon kallopterus
Valenciennea strigata
Caranx melampygus
Bothus pantherinus
Bothus mancus
Crenimugil crenilabis
Stegastes nigricans
Stegastes fasciolatus
Abudefduf septemfasciatus
Abudefduf sordidus
Abudefduf sexfasciatus
Dascyllus aruanus
Dascyllus trimaculatus
Dascyllus flavicaudus
Chromis margaritifer
Chromis atripectoralis
Chromis viridis
Pomachromis fuscidorsalis
Chrysiptera brownriggii
Pomacentrus pavo
Istiblennius edentulus
7.478
trait value
13.01
length=73.025

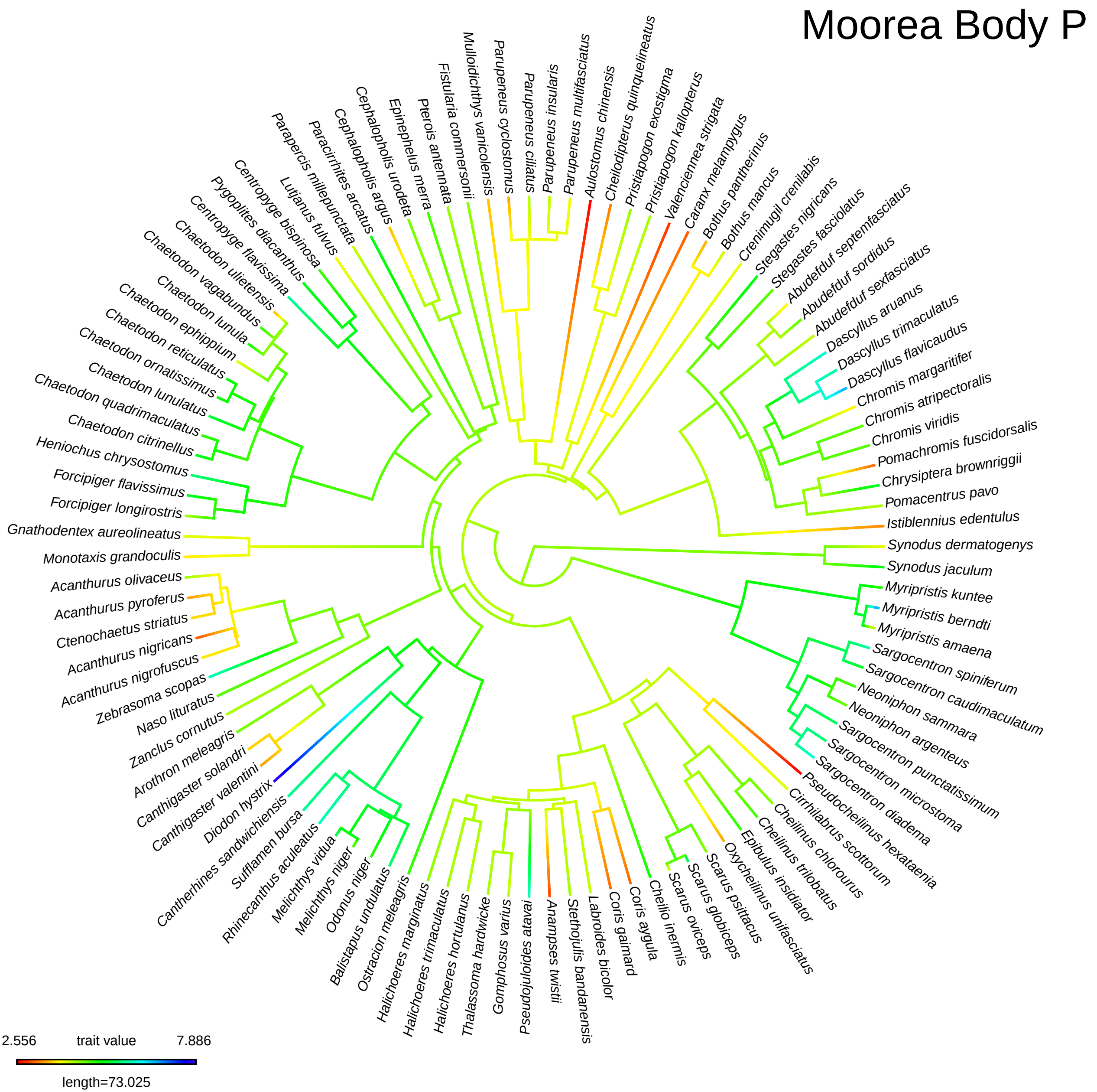

Moorea Body P
Forcipiger longirostris
Forcipiger flavissimus
Heniochus chrysostomus
Chaetodon citrinellus
Chaetodon quadrimaculatus
Chaetodon lunulatus
Chaetodon ornatissimus
Chaetodon reticulatus
Chaetodon ephippium
Chaetodon lunula
Chaetodon vagabundus
Chaetodon ulietensis
Centropyge flavissima
Pygoplites diacanthus
Centropyge bispinosa
Lutjanus fulvus
Parapercis millepunctata
Paracirrhites arcatus
Cephalopholis argus
Cephalopholis urodeta
Epinephelus merra
Pterois antennata
Fistularia commersonii
Mulloidichthys vanicolensis
Parupeneus cyclostomus
Parupeneus ciliatus
Parupeneus insularis
Parupeneus multifasciatus
Aulostomus chinensis
Cheilodipterus quinquelineatus
Pristiapogon exostigma
Pristiapogon kallopterus
Valenciennea strigata
Caranx melampygus
Bothus pantherinus
Bothus mancus
Crenimugil crenilabis
Stegastes nigricans
Stegastes fasciolatus
Abudefduf septemfasciatus
Abudefduf sordidus
Abudefduf sexfasciatus
Dascyllus aruanus
Dascyllus trimaculatus
Dascyllus flavicaudus
Chromis margaritifer
Chromis atripectoralis
Chromis viridis
Pomachromis fuscidorsalis
Chrysiptera brownriggii
Pomacentrus pavo
Istiblennius edentulus
Synodus dermatogenys
Synodus jaculum
Myripristis kuntee
Myripristis berndti
Myripristis amaena
Sargocentron spiniferum
Sargocentron caudimaculatum
Neoniphon sammara
Neoniphon argenteus
Sargocentron punctatissimum
Sargocentron microstoma
Sargocentron diadema
Pseudocheilinus hexataenia
Cirrhilabrus scottorum
Cheilinus chlorourus
Cheilinus trilobatus
Epibulus insidiator
Oxycheilinus unifasciatus
Scarus psittacus
Scarus globiceps
Scarus oviceps
Cheilio inermis
Coris aygula
Coris gaimard
Labroides bicolor
Stethojulis bandanensis
Anampses twistii
Pseudojuloides atavai
Gomphosus varius
Thalassoma hardwicke
Halichoeres hortulanus
Halichoeres trimaculatus
Halichoeres marginatus
Ostracion meleagris
Balistapus undulatus
Odonus niger
Melichthys niger
Melichthys vidua
Rhinecanthus aculeatus
Sufflamen bursa
Cantherhines sandwichiensis
Diodon hystrix
Canthigaster valentini
Canthigaster solandri
Arothron meleagris
Zanclus cornutus
Naso lituratus
Zebrasoma scopas
Acanthurus nigrofuscus
Acanthurus nigricans
Ctenochaetus striatus
Acanthurus pyroferus
Acanthurus olivaceus
Monotaxis grandoculis
Gnathodentex aureolineatus
2.556
trait value
7.886
length=73.025

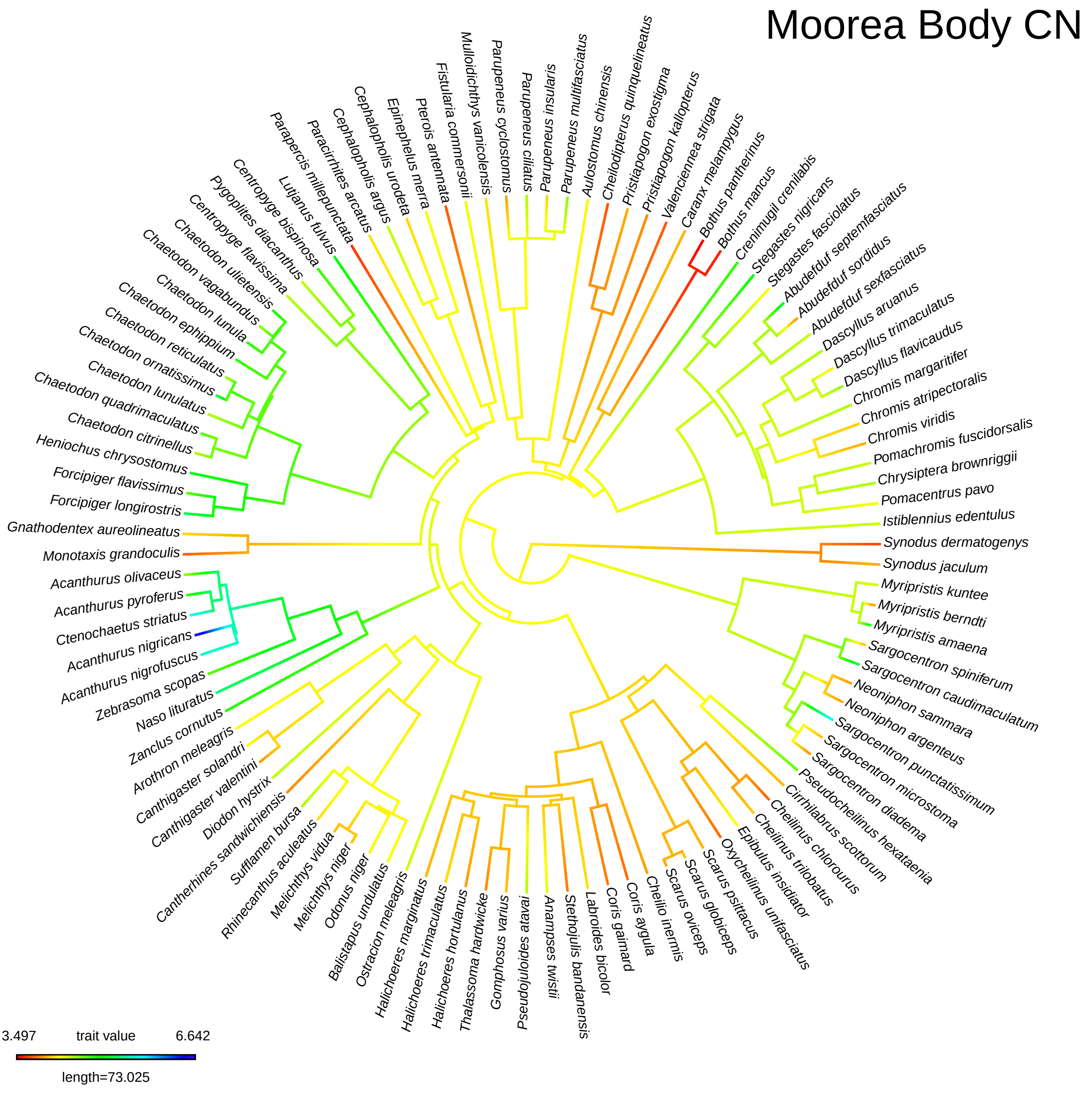
Moorea Body CN
Synodus dermatogenys
Synodus jaculum
Myripristis kuntee
Myripristis berndti
Myripristis amaena
Sargocentron spiniferum
Sargocentron caudimaculatum
Neoniphon sammara
Neoniphon argenteus
Sargocentron punctatissimum
Sargocentron microstoma
Sargocentron diadema
Pseudocheilinus hexataenia
Cirrhilabrus scottorum
Cheilinus chlorourus
Cheilinus trilobatus
Epibulus insidiator
Oxycheilinus unifasciatus
Scarus psittacus
Scarus globiceps
Scarus oviceps
Cheilio inermis
Coris aygula
Coris gaimard
Labroides bicolor
Stethojulis bandanensis
Anampses twistii
Pseudojuloides atavai
Gomphosus varius
Thalassoma hardwicke
Halichoeres hortulanus
Halichoeres trimaculatus
Halichoeres marginatus
Ostracion meleagris
Balistapus undulatus
Odonus niger
Melichthys niger
Melichthys vidua
Rhinecanthus aculeatus
Sufflamen bursa
Cantherhines sandwichiensis
Diodon hystrix
Canthigaster valentini
Canthigaster solandri
Arothron meleagris
Zanclus cornutus
Naso lituratus
Zebrasoma scopas
Acanthurus nigrofuscus
Acanthurus nigricans
Ctenochaetus striatus
Acanthurus pyroferus
Acanthurus olivaceus
Monotaxis grandoculis
Gnathodentex aureolineatus
Forcipiger longirostris
Forcipiger flavissimus
Heniochus chrysostomus
Chaetodon citrinellus
Chaetodon quadrimaculatus
Chaetodon lunulatus
Chaetodon ornatissimus
Chaetodon reticulatus
Chaetodon ephippium
Chaetodon lunula
Chaetodon vagabundus
Chaetodon ulietensis
Centropyge flavissima
Pygoplites diacanthus
Centropyge bispinosa
Lutjanus fulvus
Parapercis millepunctata
Paracirrhites arcatus
Cephalopholis argus
Cephalopholis urodeta
Epinephelus merra
Pterois antennata
Fistularia commersonii
Mulloidichthys vanicolensis
Parupeneus cyclostomus
Parupeneus ciliatus
Parupeneus insularis
Parupeneus multifasciatus
Aulostomus chinensis
Cheilodipterus quinquelineatus
Pristiapogon exostigma
Pristiapogon kallopterus
Valenciennea strigata
Caranx melampygus
Bothus pantherinus
Bothus mancus
Crenimugil crenilabis
Stegastes nigricans
Stegastes fasciolatus
Abudefduf septemfasciatus
Abudefduf sordidus
Abudefduf sexfasciatus
Dascyllus aruanus
Dascyllus trimaculatus
Dascyllus flavicaudus
Chromis margaritifer
Chromis atripectoralis
Chromis viridis
Pomachromis fuscidorsalis
Chrysiptera brownriggii
Pomacentrus pavo
Istiblennius edentulus
3.497
trait value
6.642
length=73.025

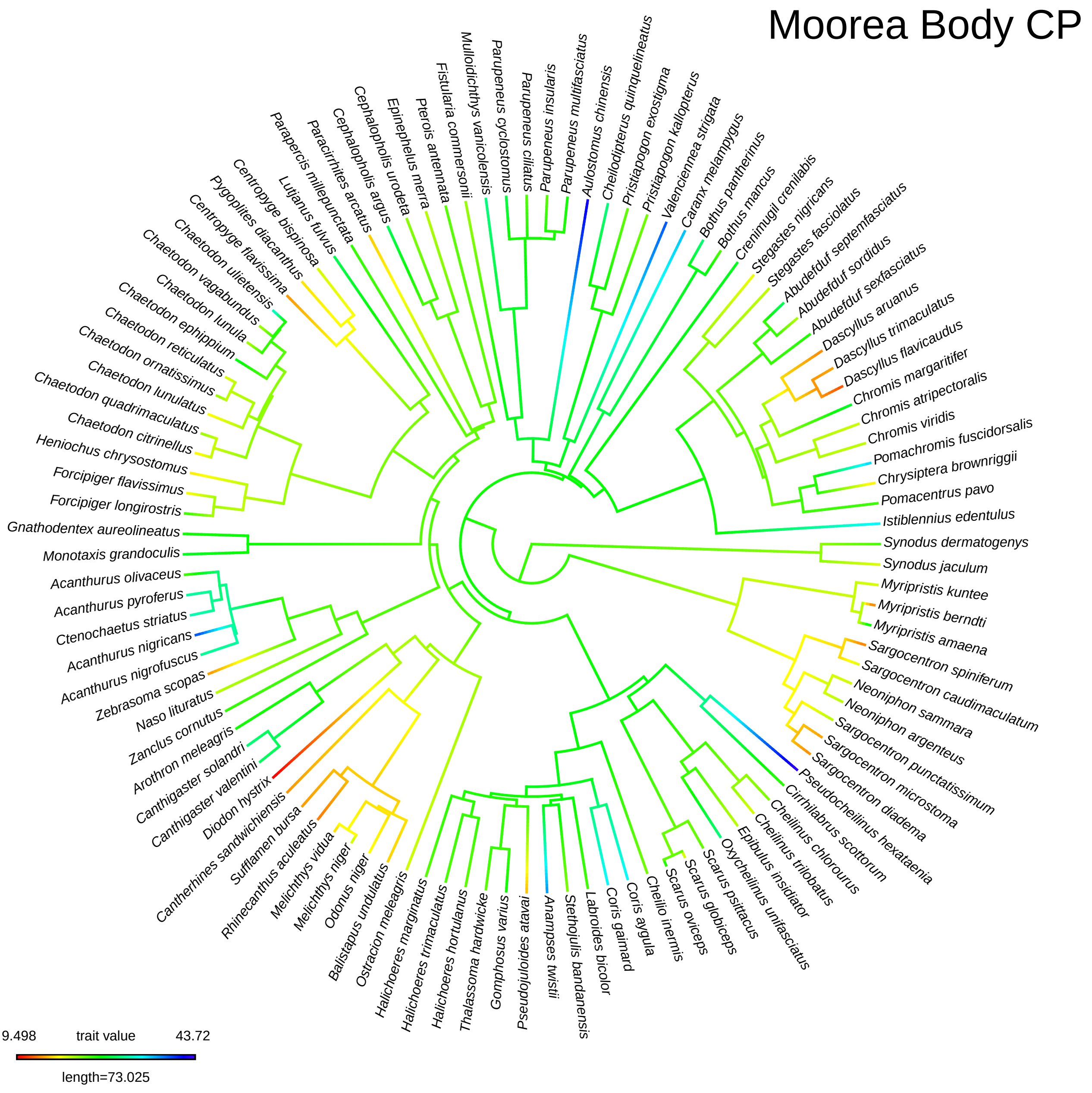

Moorea Body CP
Gnathodentex aureolineatus
Monotaxis grandoculis
Forcipiger longirostris
Forcipiger flavissimus
Heniochus chrysostomus
Chaetodon citrinellus
Chaetodon quadrimaculatus
Chaetodon lunulatus
Chaetodon ornatissimus
Chaetodon reticulatus
Chaetodon ephippium
Chaetodon lunula
Chaetodon vagabundus
Chaetodon ulietensis
Centropyge flavissima
Pygoplites diacanthus
Centropyge bispinosa
Lutjanus fulvus
Parapercis millepunctata
Paracirrhites arcatus
Cephalopholis argus
Cephalopholis urodeta
Epinephelus merra
Pterois antennata
Fistularia commersonii
Mulloidichthys vanicolensis
Parupeneus cyclostomus
Parupeneus ciliatus
Parupeneus insularis
Parupeneus multifasciatus
Aulostomus chinensis
Cheilodipterus quinquelineatus
Pristiapogon exostigma
Pristiapogon kallopterus
Valenciennea strigata
Caranx melampygus
Bothus pantherinus
Bothus mancus
Crenimugil crenilabis
Stegastes nigricans
Stegastes fasciolatus
Abudefduf septemfasciatus
Abudefduf sordidus
Abudefduf sexfasciatus
Dascyllus aruanus
Dascyllus trimaculatus
Dascyllus flavicaudus
Chromis margaritifer
Chromis atripectoralis
Chromis viridis
Pomachromis fuscidorsalis
Chrysiptera brownriggii
Pomacentrus pavo
Istiblennius edentulus
Synodus dermatogenys
Synodus jaculum
Myripristis kuntee
Myripristis berndti
Myripristis amaena
Sargocentron spiniferum
Sargocentron caudimaculatum
Neoniphon sammara
Neoniphon argenteus
Sargocentron punctatissimum
Sargocentron microstoma
Sargocentron diadema
Pseudocheilinus hexataenia
Cirrhilabrus scottorum
Cheilinus chlorourus
Cheilinus trilobatus
Epibulus insidiator
Oxycheilinus unifasciatus
Scarus psittacus
Scarus globiceps
Scarus oviceps
Cheilio inermis
Coris aygula
Coris gaimard
Labroides bicolor
Stethojulis bandanensis
Anampses twistii
Pseudojuloides atavai
Gomphosus varius
Thalassoma hardwicke
Halichoeres hortulanus
Halichoeres trimaculatus
Halichoeres marginatus
Ostracion meleagris
Balistapus undulatus
Odonus niger
Melichthys niger
Melichthys vidua
Rhinecanthus aculeatus
Sufflamen bursa
Cantherhines sandwichiensis
Diodon hystrix
Canthigaster valentini
Canthigaster solandri
Arothron meleagris
Zanclus cornutus
Naso lituratus
Zebrasoma scopas
Acanthurus nigrofuscus
Acanthurus nigricans
Ctenochaetus striatus
Acanthurus pyroferus
Acanthurus olivaceus
9.498
trait value
43.72
length=73.025

Moorea Body NP

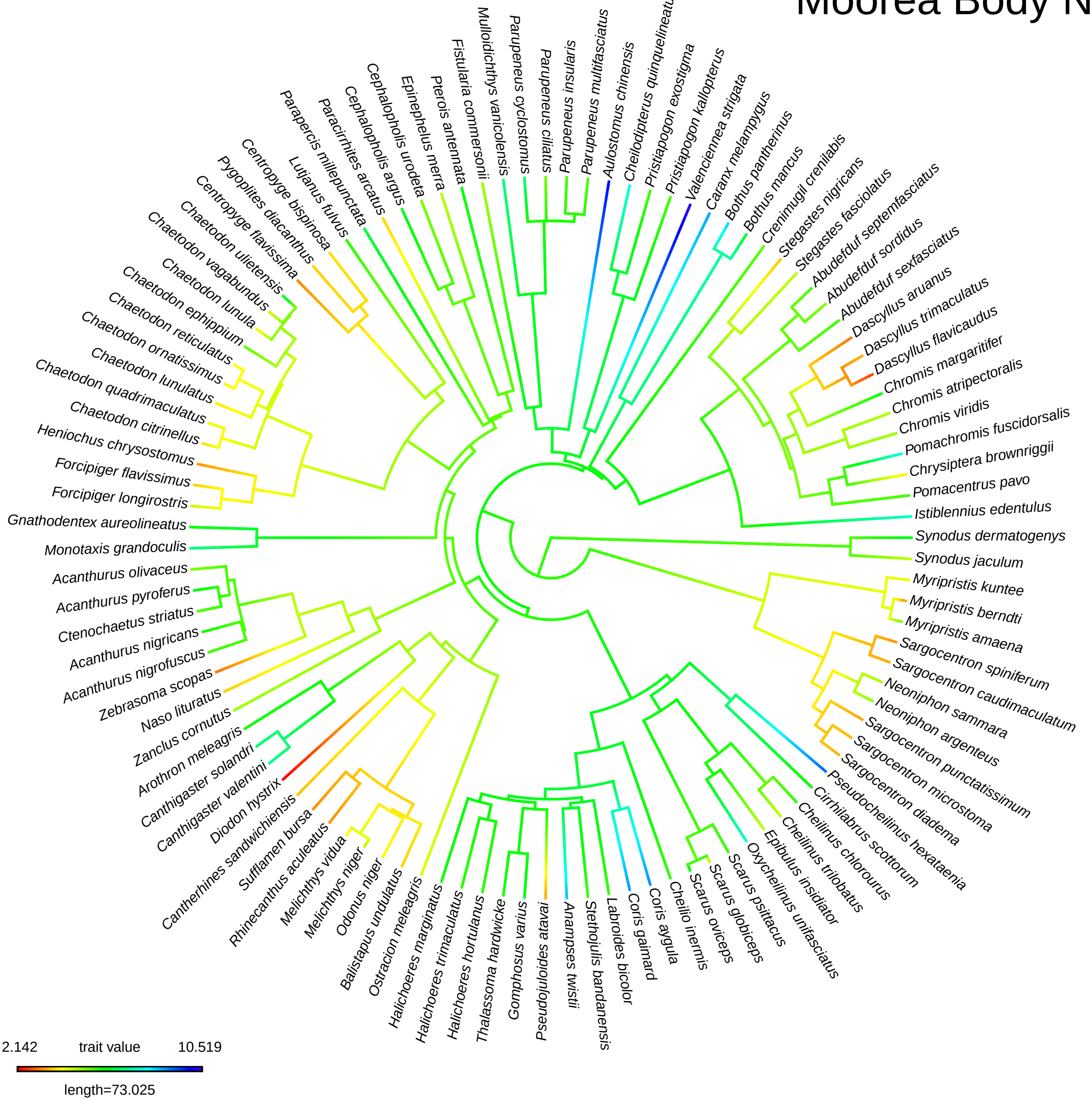

Supplement: Supplementary file 1 — Supplementary Information [file 41467_2021_25528_MOESM1_ESM.pdf]
