# Supplementary material for: Phylogenetic conservatism drives nutrient dynamics of coral reef fishes
Source: Nat Commun. 2021 Sep 14;12:5432. doi: 10.1038/s41467-021-25528-0 (PMC8440548; doi:10.1038/s41467-021-25528-0)
Supplement: Supplementary file 3 — Reporting Summary [file 41467_2021_25528_MOESM3_ESM.pdf]

nature research

Corresponding author(s): Jacob Allgeier

Last updated by author(s): Y D 2021-7-27

Reporting Summary

Nature Research wishes to improve the reproducibility of the work that we publish. This form provides structure for consistency and transparency in reporting. For further information on Nature Research policies, see our Editorial Policies and the Editorial Policy Checklist.

Statistics

For all statistical analyses, confirm that the following items are present in the figure legend, table legend, main text, or Methods section.

n/a	Confirmed	
☐	☒	The exact sample size (*n*) for each experimental group/condition, given as a discrete number and unit of measurement
☐	☒	A statement on whether measurements were taken from distinct samples or whether the same sample was measured repeatedly
☐	☒	The statistical test(s) used AND whether they are one- or two-sided<br>*Only common tests should be described solely by name; describe more complex techniques in the Methods section.*
☐	☒	A description of all covariates tested
☐	☒	A description of any assumptions or corrections, such as tests of normality and adjustment for multiple comparisons
☐	☒	A full description of the statistical parameters including central tendency (e.g. means) or other basic estimates (e.g. regression coefficient) AND variation (e.g. standard deviation) or associated estimates of uncertainty (e.g. confidence intervals)
☐	☒	For null hypothesis testing, the test statistic (e.g. *F*, *t*, *r*) with confidence intervals, effect sizes, degrees of freedom and *P* value noted<br>*Give P values as exact values whenever suitable.*
☐	☒	For Bayesian analysis, information on the choice of priors and Markov chain Monte Carlo settings
☐	☒	For hierarchical and complex designs, identification of the appropriate level for tests and full reporting of outcomes
☐	☒	Estimates of effect sizes (e.g. Cohen's *d*, Pearson's *r*), indicating how they were calculated

Our web collection on statistics for biologists contains articles on many of the points above.

Software and code

Policy information about availability of computer code

Data collection	All data was collected in the field. Fishtree package in R was used for phylogenetic data collection.
Data analysis	All data was analyzed using R software, including fishtree and MCMCGlmm packages.

For manuscripts utilizing custom algorithms or software that are central to the research but not yet described in published literature, software must be made available to editors and reviewers. We strongly encourage code deposition in a community repository (e.g. GitHub). See the Nature Research guidelines for submitting code & software for further information.

Data

Policy information about availability of data

All manuscripts must include a data availability statement. This statement should provide the following information, where applicable:

- Accession codes, unique identifiers, or web links for publicly available datasets
- A list of figures that have associated raw data
- A description of any restrictions on data availability

All data and code will be made publicly available on GitHub account (https://github.com/Allgeier-Lab) shortly after publication. Data for the Caribbean are in press as a datapaper in Ecology (Allgeier in press Ecology).

Field-specific reporting

Please select the one below that is the best fit for your research. If you are not sure, read the appropriate sections before making your selection.

☐ Life sciences ☐ Behavioural & social sciences ☒ Ecological, evolutionary & environmental sciences

For a reference copy of the document with all sections, see nature.com/documents/nr-reporting-summary-flat.pdf

Ecological, evolutionary & environmental sciences study design

All studies must disclose on these points even when the disclosure is negative.

Study description	The study captured fishes from coastal ecosystems of The Bahamas, and French Polynesia. Bioassays were then conducted on the fishes to determine their excretion. Fishes were then euthanized, dried, and analyzed for body nutrient content. Theses data were then analyzed as explained in the manuscript. The field sampling was opportunistic and our goal was to collect as many species, and individuals within species as possible, ideally across the range of trophic groups within both systems.
Research sample	We opportunistically sampled as many fishes from The Bahamas and French Polynesia as we were able to capture within the allotted time of our field work.
Sampling strategy	We collected as many individuals as we were able to.
Data collection	Data were collected by JEA, KSM, and DEB and were recorded by all three participants. See manuscript for full details.
Timing and spatial scale	Samples were collected in The Bahamas by JEA from 2008-2011 in the spring months from January - May. Samples were collected from a single large embayment - The Bight of Old Robinson. Samples were collected in French Polynesia by JEA, KSM, and DEB in 2015-2016 in the austral winter months of June and July and were collected opportunistically on fringing reefs, back reefs, and fore reefs depending on where species persist.
Data exclusions	Excretion data was excluded from the analysis when it was below 0.002 ug/L and 0.02 ug/L per hour for phosphorus and nitrogen, respectively. These values are within the range of analytical error and were determined to be compromised samples.
Reproducibility	In all cases we ran multiple standard curves when analyzing water chemistry, multiple standards when analyzing body nutrient content. Methods for measuring excretion in the field have been extensively vetted by the authors (see Allgeier et al. 2013, 2014, 2015a, 2015, 2016, 2020). Attempts to repeat experiments were successful.
Randomization	In all cases the order by which individual fishes were placed in the experimental chambers for excretion was random.
Blinding	*N/A*

Did the study involve field work? ☒ Yes ☐ No

Field work, collection and transport

Field conditions	Field conditions are tropical coastal ecosystems. In both cases research was conducted in the dry season.
Location	Abaco Island, The Bahamas, and Mo'orea French Polynesia
Access & import/export	In all cases samples were exported with permission from the respective governments (The Bahamas, and Mo'orea French Polynesia).
Disturbance	Disturbances to the ecosystems may have been caused by fishing. In all cases we took all precautions to minimize these effects.

Disturbances were minimized by not fishing intensively in any single location

Reporting for specific materials, systems and methods

We require information from authors about some types of materials, experimental systems and methods used in many studies. Here, indicate whether each material, system or method listed is relevant to your study. If you are not sure if a list item applies to your research, read the appropriate section before selecting a response.

Materials & experimental systems

n/a	Involved in the study
☒	☐ Antibodies
☒	☐ Eukaryotic cell lines
☒	☐ Palaeontology and archaeology
☐	☒ Animals and other organisms
☒	☐ Human research participants
☒	☐ Clinical data
☒	☐ Dual use research of concern

Methods

n/a	Involved in the study
☒	☐ ChIP-seq
☒	☐ Flow cytometry
☒	☐ MRI-based neuroimaging

Animals and other organisms

Policy information about studies involving animals; ARRIVE guidelines recommended for reporting animal research

Laboratory animals	*N/A*
Wild animals	Coral reef fishes were caught using hook and line, nets, and traps. In all cases fishes were transported back to the field station where the bioassays were conducted for excretion. Fishes were euthanized immediately after excretion trials took place in a humane way and immediately frozen. See supplemental material Table S1 for details of fishes collected for this study.
Field-collected samples	*N/A*
Ethics oversight	All research protocols were vetted by the (at the time) sponsoring institution - the University of Georgia for the work in The Bahamas (AUP # A2009-10003-0), and University of California Santa Barbara for the work in Mo'orea (IACUC #915 2016-2019).

Note that full information on the approval of the study protocol must also be provided in the manuscript.
